# Splicing imbalances in basal-like breast cancer underpin perturbation of cell surface and oncogenic pathways and are associated with patients’ survival

**DOI:** 10.1038/srep40177

**Published:** 2017-01-06

**Authors:** Filipe Gracio, Brian Burford, Patrycja Gazinska, Anca Mera, Aisyah Mohd Noor, Pierfrancesco Marra, Cheryl Gillett, Anita Grigoriadis, Sarah Pinder, Andrew Tutt, Emanuele de Rinaldis

**Affiliations:** 1 Guy’s and St Thomas’ NHS Foundation Trust and King’s College London NIHR Biomedical Research Centre – Translational Bioinformatics Platform, Guy’s Hospital, London, UK; 2 Breast Cancer Now Research Unit King’s College London, School of Medicine, Division of Cancer Studies, Bermondsey Wing, Guy’s Hospital, London; 3 Research Oncology, King’s College London, School of Medicine, Division of Cancer Studies, Bermondsey Wing, Guy’s Hospital, London, UK; 4 Cancer Epidemiology Group, King’s Health Partners AHSC, King’s College London, School of Medicine, Division of Cancer Studies, Bermondsey Wing, Guy’s Hospital, London, UK; 5 King’s Health Partners Cancer Biobank, King’s College London Faculty of Life Sciences & Medicine, Division of Cancer Studies, Bermondsey Wing, Guy’s Hospital, London, UK; 6 Breast Cancer Now Toby Robins Research Centre, Institute of Cancer Research, 237 Fulham Road, London

## Abstract

Despite advancements in the use of transcriptional information to understand and classify breast cancers, the contribution of splicing to the establishment and progression of these tumours has only recently starting to emerge. Our work explores this lesser known landscape, with special focus on the basal-like breast cancer subtype where limited therapeutic opportunities and no prognostic biomarkers are currently available. Using ExonArray analysis of 176 breast cancers and 9 normal breast tissues we demonstrate that splicing levels significantly contribute to the diversity of breast cancer molecular subtypes and explain much of the differences compared with normal tissues. We identified pathways specifically affected by splicing imbalances whose perturbation would be hidden from a conventional gene-centric analysis of gene expression. We found that a large fraction of them involve cell-to-cell communication, extracellular matrix and transport, as well as oncogenic and immune-related pathways transduced by plasma membrane receptors. We identified 247 genes in which splicing imbalances are associated with clinical patients’ outcome, whilst no association was detectable at the gene expression level. These include the signaling gene TGFBR1, the proto-oncogene MYB as well as many immune-related genes such as CCR7 and FCRL3, reinforcing evidence for a role of immune components in influencing breast cancer patients’ prognosis.

Breast cancer is a heterogeneous disease that comprises tumour subgroups with substantial differences in biology, clinical outcomes and responses to treatment. Whilst the debate on the most appropriate definition of breast cancer subtypes is still open, it is now accepted that breast cancer consists of at least five different molecular subtypes which include - according to the PAM50 classification scheme^1^ - *basal-like, HER2, Luminal A, Luminal B* and the additional category of *Normal-like*, made of tumours which transcriptionally resemble normal breast tissue samples^2^. These molecular subtypes have clear, although not complete, correlation with clinically defined tumour classes, based on the histological assessment of the oestrogen (ER) and progesterone (PR) receptors and human epidermal growth factor receptor 2 (HER2). Basal-like breast cancers overlap to a large degree with clinically defined triple-negative tumours (ER-negative, PR-negative and HER2-negative), whilst Luminal A/B and HER2 correspond respectively to ER negative and ER-negative/HER2-positive tumours. What makes the discovery and exploration of these subtypes relevant is the evidence of their association with different clinical outcomes, ranging from the best-prognosis Luminal A tumors to poor prognosis HER-2 and basal-like tumors, as well as underlying differences in biology reflected in different patterns of response to therapeutic agents^3^.

In the last decade, genomics analyses have significantly improved our knowledge of breast cancer. Extensive and integrated molecular studies of increasing size and resolution are revealing the existence of additional tumour subgroups with distinct molecular properties^4^,^5^,^6^,^7^. However, only limited information is currently available on the role of alternative splicing in the establishment and progression of these tumours, and on the contribution of splicing to breast cancer heterogeneity and its potential for biomarker development^8^,^9^.

Alternative splicing is a key post-transcriptional mechanism affecting more than 90% of human genes and is responsible for the generation of protein isoforms with very different biological properties and functions^10^,^11^. Antagonistic splice variants of genes involved in differentiation, apoptosis, invasion and metastasis often exist in a delicate equilibrium that is found to be perturbed in tumours. Indeed, a number of studies have demonstrated that changes in splicing during cancer development alter hallmarks of cancer metastases such as cell morphology, adhesion, migration, apoptosis and proliferation processes, and that oncogenes are inactivated by alternative splicing in normal differentiation^12^.

To have an insight into the molecular perturbations induced by splicing imbalances in breast cancer we have used the Affymetrix GeneChip Exon 1.0 ST platform to analyse a well characterised patient cohort encompassing 176 samples composed primarily of tumours classified as basal-like according to PAM50^13^,^14^. This technology allows for expression profiling of individual exons and has already been applied in several cancer studies to assess transcriptional splicing variants^15^,^16^,^17^,^18^.

The exon-level resolution allowed for the measurement of the relative abundances of the exons - and therefore indirectly of the underlying isoforms - transcribed from each gene, a concept we referred to as gene’s *splicing balance*. Results reveal that an additional layer of transcriptional diversity between tumours and normal breast tissues and between different tumour molecular subtypes exists based on genes’ splicing imbalances, which goes beyond what has been observed so far in breast cancer by measuring overall gene expression levels^2^. We have attempted to quantify and to qualify this layer, investigating on the pathways affected by splicing imbalances and on the use of this information to identify therapeutic targets and clinical prognostic biomarkers.

## Results

### Samples data and clinical and molecular classification

The study is based on the analysis of Affymetrix GeneChip Exon 1.0 ST data from a set of 176 invasive breast carcinomas extracted from an equivalent number of patients, and an additional group of 9 normal breast tissues (hereby referred to as NBT samples) extracted from mammary reductions of unrelated individuals. The same cohort was analyzed in previous studies by our group^13^,^14^. Of the 176 tumours analysed, 148 were immunohistochemically ER-negative, 93 being also triple-negative. Molecular characteristics of tumour samples were analysed in association with clinical and pathological information (Additional File 1). In addition to the assignment to clinical subgroups based on ER, PR and HER2 status, tumour samples were classified according to the five intrinsic molecular subtypes (basal-like, luminal A, luminal B, HER2 and normal-like). For this we used the expression of predefined intrinsic gene lists according to the PAM50 centroid-based classification method^1^. In line with our previous analyses on the same data set^13^,^14^, triple-negative breast cancers were found to correspond mostly with the basal-like tumours (84%) while ER-positive lesions corresponded to luminal A and B subtypes (79%) (Additional File 2).

### Differential expression and differential splicing across normal tissues and breast cancer molecular subtypes

**Figure 1:**
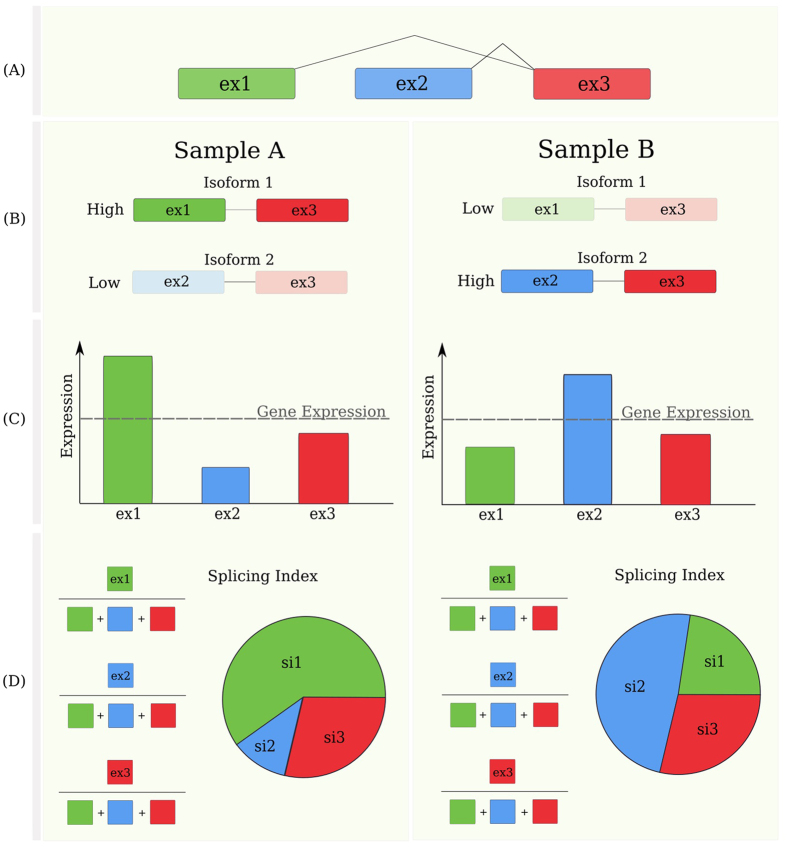
Exon expression, gene expression and splicing balance values of an exemplary three-exons gene in two samples. Panel (A): a multi exon gene model. Panel (B): illustration of a case of two samples expressing the gene with different balances of exons. Panel (C): Gene and exon level measures of expression. In the example, the two samples have equal gene expression (GE), but different splicing balances, as detected by different exon-level contributions to the overall gene expression (**D**): The Splicing Index (SI) metric used to quantify splicing imbalances is explained, showing the different contribution of each exon to the total gene expression. Note that SI, unlike exon expression, is invariant to total gene expression. This exemplifies how SI captures the splicing balance level of information, different than gene or exon expression.

We compared different breast cancer subtypes between themselves and with respect to NBT samples, on the basis of three different measurements: (i) the overall expression of genes (GE), in which multiple probes on different exons are summarised into a cumulative expression value for all transcripts of the same gene; (ii) the expression of individual exons (EE), inferred from exon-specific probes; (iii) the exon splicing levels, as measured by the splicing index (SI) metric (see methods)^19^. This metric captures the contribution of each exon to the overall expression of a gene. Differences in an exon’s SI between two sample groups reflect indeed different inclusion or exclusion rates of that exon with respect to the overall gene expression, and thus different splicing balances between the two groups (Fig. 1).

First we computed coefficients of determination using the GE/EE/SI values to assess the overall degree of similarity among tumours from the same and from different subtypes. As expected, we observed pairwise correlation levels to be significantly higher when calculated from within- then from between-subtypes. These differences are very high when GE and EE values are used, and lower when using SI values, in keeping with the fact that subtypes are defined based on overall gene expression and not splicing information (Additional File 3).

Then we compared tumour subtypes between themselves and with normal breast tissues using GE/EE/SI metrics. In this way it was possible to reveal different splicing balances, in the presence or absence of whole-gene differential expression, thus adding a layer of resolution to standard gene-centric transcriptional analyses.

By analysing the deviation of the obtained distribution of p-values for each comparison from the distribution generated under the null hypothesis of no average differences we could then infer the overall diversity between groups, due to each of these measures respectively, along with their statistical significance (see Materials and Methods).

The observed pairwise differences are beyond what would be observed by random fluctuations under the null hypothesis, thus pointing to their internal statistical significance (Additional File 4).

Having established the principle that splicing imbalances contribute to overall breast cancer diversity and to the differences between tumour and NBT samples, we then tried to quantify and to define the borders between gene expression and splicing imbalance effects.

**Figure 2:**
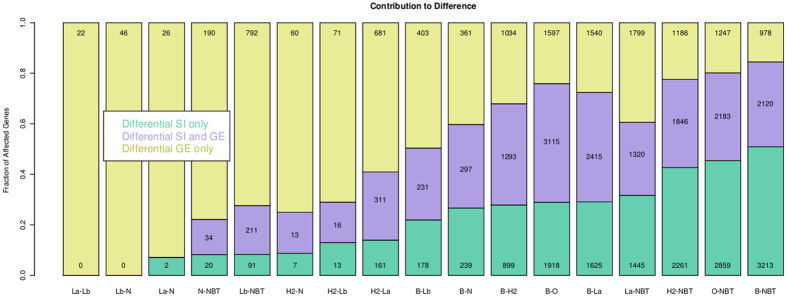
Pairwise transcriptional differences between tumour subtypes and NBT. Each bar represents the results of comparison between two groups of samples. Key: B = basal-like, H2 = HER2, La = LuminalA, Lb = LuminalB, N = Normal like, O = Others (non-basal-like rumour), NBT = normal breast tissue. The relative fractions of genes differentially expressed, with differential splicing balances or both are reported in different colours. The absolute numbers of genes falling into each of these categories is also reported.

In all comparisons we could identify, along with genes showing both differential expression and splicing balance (GE and SI overlaps), also genes having differential splicing balances but not overall differential expression (GE and SI disjunctions) (Fig. 2). Perturbation of these genes would not have been detected by looking at GE values alone. By quantifying GE/SI overlaps and disjunctions we could therefore assess in each comparison the GE and SI relative contributions to the overall transcriptional diversity across different sample groups: on one extreme is the Luminal A/Luminal B pair, whose differences are mainly explained by GE levels; on the other is the basal-like tumours and NBT pair, showing marked differences both in GE and SI levels. Noticeably, whilst the absolute number of genes differentially expressed and spliced might differ from pair to pair due to different samples sizes, their percentage contribution to the overall set of perturbed genes is stable and independent of both sample sizes and the q-value thresholds used for statistical significance.

These results indicate the distinct value of looking at differential splicing in addition to differential expression and demonstrate that splicing mechanisms significantly contribute to the diversity across tumour subtypes and to their differences with respect to normal breast tissue counterpart.

On this basis we also investigated the value of splicing data for potential application to molecular diagnostics and tumour subtype classification. We adopted a decision-tree algorithm of classification, seeking to identify basal-like tumours, from a pool of basal-like tumour and NBT samples – either by using GE, EE, SI, or using SI after having filtered out genes differentially expressed between basal-like tumours and NBT samples (see Materials and Methods). Results show that not only GE data but also SI information, used as the sole input data, is capable of correctly classifying tumour samples, with a performance of over 90% specificity and 70% sensitivity using 1000 randomly selected genes (Additional File 5, see also Methods). The ability of SI information to distinguish between sample types, was also confirmed by unsupervised clustering, based on principal component analysis (PCA) (Additional File 6).

### Comparative assessment of splicing-level results

The validity of our results was assessed through comparison with three independent studies, respectively on a panel of breast cancer cell lines using exon array-based method^8^, on a small group of triple-negative primary breast cancers using RNA sequencing-based technology^9^ and on a larger RNA sequencing data set of basal-like breast tumours and NBT from The Cancer Genome Atlas (TCGA: http://cancergenome.nih.gov/). In all three cases we compared the list of genes found to have differential splicing balances in our data (based on differential SI) with the equivalent list in the external data set.

Comparison with the array-based cell line study analysis^8^ showed a significant overlap, with 21 out of 58 genes differentially spliced between basal-like vs luminal cell lines confirmed in our study (45% of overlap, Fisher test p-value < 10^−4^) (Additional File 7). The second check against the triple-negative (n = 6) *vs*. normal breast tissues (n = 3) analysis carried out using RNA sequencing technology^9^ produced also a very significant overlap, with 121 out the 371 genes identified in this study to be differentially spliced confirmed by our results (32% of overlap, Fisher test p-values respectively <10^−6^) (Additional File 7).

The third data encompassed 92 basal-like tumours and 133 NBT samples and was used to run a more thorough comparison, where genes were selected for being differentially spliced but not differentially expressed in basal-like tumours vs NBT in both data sets. Out of 1.822 identified in the external data set to fulfill these criteria, 408 were confirmed by our study (22% of overlap, Fisher test p-value < 10^−12^) (Additional File 7).

We also looked for experimental evidences in support of the differential splicing observed in our data set between basal-like tumours and NBT samples. The 100 genes with the lowest p-values for differential splicing balance between basal-like tumours and NBT samples in our data were selected and used for automated literature searches to explore experimental evidences in support of our findings.

Several of them had previously been reported to have breast cancer specific splicing events or differential isoform expression. Examples are FANCD2, RAD54, BIRC5 (survivin) and ASF1B^20^,^21^,^22^,^23^,^24^,^25^,^26^,^27^. Others amongst our list were detected to be differentially spliced in other forms of cancer, or cancer cell lines: FoxM1 CDKN3, ZBTB16, AURKB, CHEK1, SGOL1, SULF1, CDC45 and UBE2C^28^,^29^,^30^,^31^,^32^,^33^,^34^,^35^,^36^,^37^,^38^,^39^. Other cases had been shown to have cell cycle dependent isoform expression. These are: KIF18A, NEK2, MKI67 and CCNA2^40^,^41^,^42^,^43^,^44^. By taking a complementary approach and looking at genes previously shown to be differentially spliced in breast cancer we also observed a high level of concordance, for example Tenascin C, CD44, CD47, RELA, PTK2, ESR1, SYK, BRCA1, LARP1 and ADD3^9^,^45^,^46^,^47^,^48^,^49^,^50^,^51^,^52^.

The convergence of our results with these independent genomic studies and experimental evidences pointed to the overall reliability of our results and provided the basis for further downstream analyses.

### Experimental validation of differential splicing results

To provide experimental support to our findings, a selection of genes showing differential splicing was assayed on a Bioanalyzer DNA7500 after RT-PCR based amplification (Additional File 8). We have selected 11 genes to be either differentially spliced between basal-like tumours and normal samples or differentially spliced in basal-like tumours, between patients with respectively better and worse outcome. Amplified full length cDNAs from each gene were size separated on a Bioanalyzer DNA7500 (see Materials and Methods), allowing comparison between the patterns of transcriptional isoforms expressed in two groups of tumours. Among the 11 genes selected we could successfully amplify 9 of them, of which 8 differentially spliced between basal-like tumours and normal samples (AURKA, AURKB, BCL2-a, NEK2, RRM2, TGFBR1, UBE2C, ZBTB16) and 2 differentially spliced in basal-like tumours, between patients with respectively better and worse outcome (CCR7, TGFBR1). Results obtained for these genes confirm differential splicing, with one of more isoforms of each gene showing differential expression between the two compared groups (Additional File 8).

The analyses on this small gene panel served as a proof of concept to validate our methodological framework for identification of genes undergoing differential splicing, based on the Affymetrix Exon Array 1.0 ST arrays, a platform which has also been extensively validated elsewhere^18^,^53^,^54^,^55^.

### Cell functions and pathways affected at splicing level in basal-like tumours

**Figure 3:**
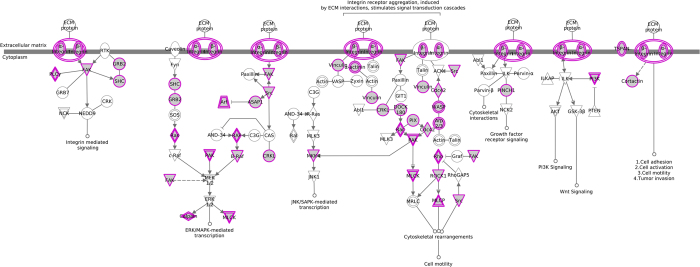
The integrin signalling pathway (Ingenuity^®^ Systems). In purple are genes affected by splicing imbalances between basal-like tumours vs NBT, with no evidence of whole-gene differential expression.

We aimed to identify the cellular functions and pathways altered as a consequence of differential splicing balances in basal-like tumours, as compared to NBT samples. We started from the list of genes showing differential splicing balances but not differential expression between these two groups and evaluated the affected pathways using gene set enrichment and Ingenuity-based analyses^56^. We observed that in basal-like tumours splicing imbalances determine, or contribute to the deregulation of many key cancer “hallmarks”^57^. These include known oncogenes (BCL2, BRAF), caspases (CASP6/7), transcription factors (E2F3), cell cycle genes (CDC42, CDK2, CDKN2A), cancer related kinases (JAK2/3, MAPK4/6/14) and DNA repair genes (PARP1, RAD50 and BRCA1). Moreover, we found a clear enrichment for cell surface and extracellular matrix genes, controlling cellular adhesion and cellular motility. Equally striking is the enrichment for oncogenic signaling pathways, mediated by cell surface receptors (complete results are listed in Additional File 9). In these cases surface receptors as well as downstream intracellular signaling proteins showed splicing level imbalances. Examples are the integrin and paxillin signalling pathways which emerged with highest ranking. These membrane mediated pathways are involved in cellular spreading, cell motility and cancer development^58^ and exert their function by transducing the extracellular signal to key oncogenic pathways such as *MAPK/ERK, Wnt, Rho, mTOR, PTEN* and *PI3K/AKT* signaling pathways (Fig. 3 and Additional File 10).

**Figure 4:**
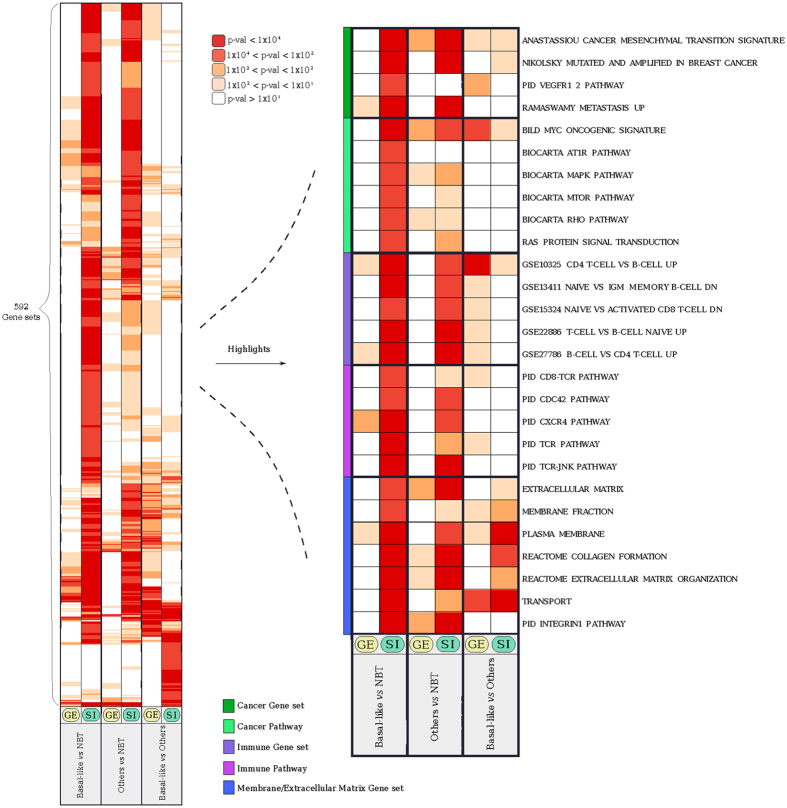
Heatmap of gene sets and pathways specifically perturbed by splicing imbalances. Each column represents a pairwise comparison (either at GE or SI level), each row is a gene set or a pathway, and color-coded is the significance level of the enrichment. On the left are represented the complete results of the analysis. On the right, a selection of specific gene sets and pathways is highlighted showing specific perturbations related to cancer, immune system, and transport and membrane. All reported gene sets and pathways are significantly enriched in at least one of the three pairwise comparisons illustrated. Gene sets are grouped in five different classes as indicated by the side colour bar.

We also checked whether these pathways would have emerged from standard whole-gene expression levels. To this aim we carried out a parallel gene set enrichment analysis of the two gene lists respectively based on differential gene expression (GE), and differential splicing index (SI) between basal-like breast cancer and NBT samples. We found that many of the described perturbations were specifically affected by splicing imbalances and would have been missed or largely underestimated had the same samples been analyzed at a whole-gene expression perspective. Examples include the integrin signalling pathway mentioned above, the *VEGFR1* pathway and the oncogenic *MAPK/ERK, mTOR* and *RAS* signaling pathways, which also appear to be perturbed exclusively at the splicing level (Fig. 4 and Additional File 11, Additional File 9 for complete results).

We also observed basal-like splicing specific enrichments in other sets of genes related to breast cancer. These include a cancer mesenchymal transition signature (“ANASTASSIOU CANCER MESENCHYMAL TRANSITION SIGNATURE”), genes up-regulated in metastatic breast cancer (“RAMASWAMY METASTASIS UP”) as well as genes found mutated and amplified (“NIKOLSKY MUTATED AND AMPLIFIED IN BREAST CANCER”) (Fig. 4). Other interesting enrichments relate to the perturbation of immune-related pathways, such as those mediated by *CD8, TCR, CDC42* and *JNK*, as well as sets of genes previously found to be perturbed in different types of immune cells (e.g. “CD4 T-CELL VS B-CELL UP”) (purple group in Fig. 4).

The same approach was used to explore the differences between tumour molecular subtypes. Despite the limited sample size of the non-basal-like groups (resulting into diminished statistical power) we could identify splicing-specific differences between basal-like and Luminal and HER2 subtypes, with plasma membrane receptors showing up again as specifically deregulated at splicing levels in basal-like tumours (complete results are listed in Additional File 9).

### Splicing and association with breast cancer survival

As the next step we explored the possible associations between gene splicing balances – as measured by SI - and disease outcome, using patients’ breast cancer specific survival as the clinical end point (see Materials and Methods). Keeping the same analytical framework described, we ran parallel and independent analyses using GE, EE and SI data as the predictor variables in Cox-regression univariate model, followed by Wald test. We observed that the distributions of the p-values obtained from the three analyses significantly deviate from uniform distributions, indicating that – at a general level - all these three measures, GE, EE and SI, hold statistically significant prognostic information (Additional File 12). External validation of our gene-level survival analysis results came from comparison with a large public database of Affymetrix-based tumour gene expression data, herewith referred to as the *KMP* database^59^. Out of the 204 genes associated with basal-like prognosis in our dataset (q-val < 0.1), 168 (83%) had q-val < 0.1 in the KMP database (Fisher-test p-value of the overlap <10^−20^). As a negative control, when we took the 204 genes with lowest association with prognosis from our dataset, only 25% had a q-value < 0.1 in the KMP database (a more extensive description of the comparative validation of our survival results can be found in Additional File 13).

**Figure 5:**
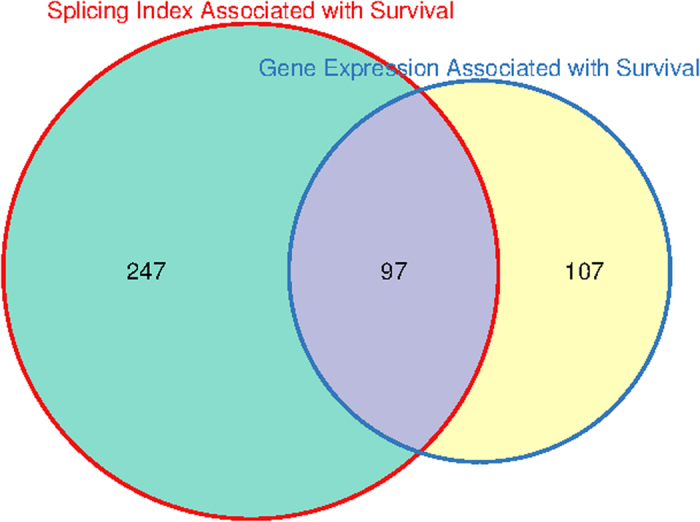
Association with breast cancer specific survival. Number of genes where GE or SI are associated with breast cancer specific survival in basal-like tumour patients.

Interesting patterns emerged from the gene-by-gene comparative analysis of the results obtained by using the GE and SI metrics. We found a total number of 344 genes whose respective splicing index values are associated with survival. Of these, 97 genes were found to have both GE and SI levels associated with survival (Fig. 5 and Additional File 14 for complete results). Examples are CYFIP2, WIPF1 and SLAMF1 (Additional Files 15–17). For these genes the overall gene expression levels - comprising the sum of all transcriptional isoforms - is associated with survival, and at the same time the splicing balance relative to one exon - that is, the contribution to the overall gene expression of one particular exon and its related transcript isoforms - is also associated with survival.

**Figure 6:**
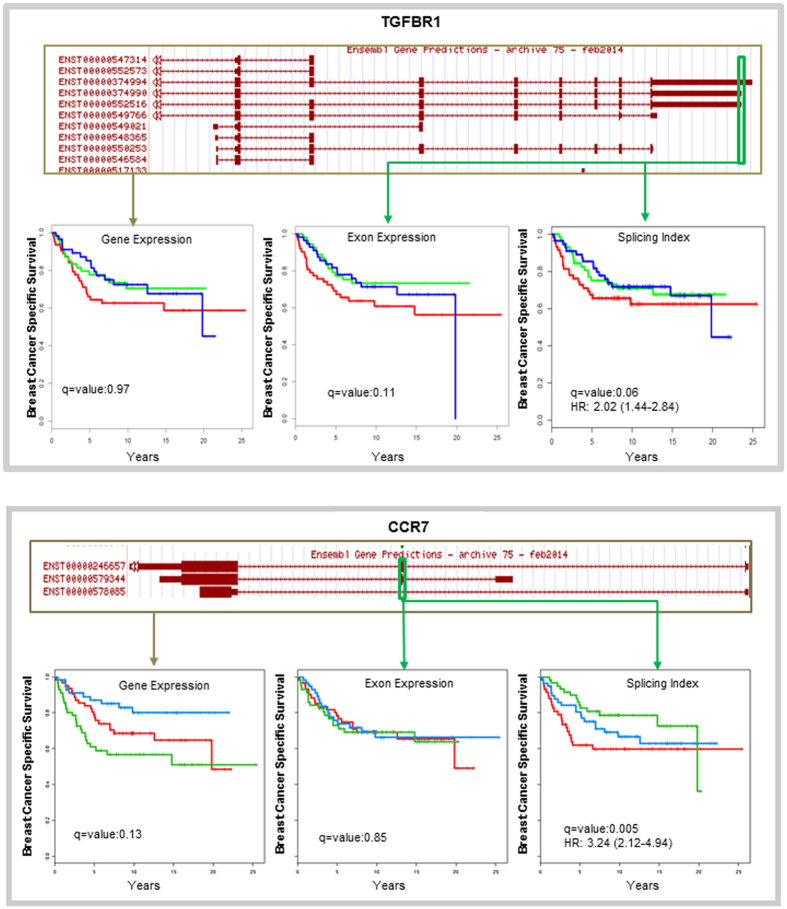
Gene models and Kaplan–Meier curves. CCR7 and TGFBR1 genes and their association with breast cancer specific survival. For each panel, on the top is a schematic representation of the gene model (from the UCSC Genome Browser). Highlighted in green are probes where the SI could be associated with survival.On the bottom are Kaplan-Meier breast cancer specific survival curves for Gene Expression, Exon Expression, and Splicing Index. In each plot, the three lines represent the top tertile (red), middle tertile (blue), and lower tertile (green) for the value of the variable. q-values for association with survival, and the hazard ratio with 95% confidence intervals are reported.

A more intriguing pattern is represented by the 247 genes whose overall expression does not show association with survival, whilst the splicing balance (as determined by the SI) of one of its exons is. In these cases what drives the association with survival is not the expression of a gene as a whole, but instead the relative abundance of the transcriptional isoforms containing a given exon. We describe two examples of genes following this pattern: CCR7 and TGFBR1 (Fig. 6). Others include proto-oncogenes such as MYB and immune-related genes such as FCRL3.

We, as well as others, have shown in previous studies that the percentage of lymphocytic infiltration represents an important prognostic factor in basal-like and triple-negative breast cancer^13^,^60^,^61^. In order to assess whether the observed associations of genes splicing balance with clinical outcome are prognostic factors independent of lymphocytic infiltration we moved to a multivariate Cox-regression model, which included this as an additional predictor variable (see Materials and Methods). Our results indicate that if lymphocytic abundance is taken into account, the significance for association with survival obtained using GE, EE, and SI gene levels is lost or significantly reduced for most of the genes (Additional File 18). In other words, transcriptional information does not contribute much to the prediction of clinical outcome when lymphocytic abundance is also available.

Taken together these data suggest that genes associated with survival in univariate analysis act in the model as a surrogate for the abundance of lymphocytic infiltration, implying that these genes are expressed in lymphocytic cells.

To investigate on this hypothesis, we examined in more details genes whose total expression levels or splicing balances were associated with patients’ survival in univariate analysis (respectively 204 and 344 genes). We observed that a significant proportion - respectively 28 and 37 - were indeed genes specifically expressed in lymphocytes - as determined by using an external transcriptional data set - or annotated to play a role in the interaction between epithelial and immune tumour compartments (see Materials and Methods and Additional File 18). Notwithstanding, we also identified two (SCGB2A1, SCGB1D1) and seven genes (TGFBR1, CD3E, UHRF1BP1L, SBF2, CCDC121, SETD8, NUCB2) whose respectively gene expression (GE) and splicing balance (SI) retain prognostic value independently of the level of lymphocytic infiltration (see Additional File 14 for complete results). Of note, the transforming growth factor TGFBR1 is a membrane protein receptor involved in many cancers and whose polymorphisms were previously observed to be associated with risk for several forms of cancer, in particular breast cancer^62^.

## Discussion

In this work we have analyzed exon level expression data of 176 breast cancer tissue and 9 non-tumour breast samples, with the aim of detecting splicing imbalances occurring in breast cancer subtypes and inferring their functional and prognostic significance.

First, we characterized splicing-driven differences across breast cancer molecular subtypes and in comparison with normal breast tissues (NBT).

Parallel analyses of whole-gene and exon-level measurements run across different group pairs showed that in addition to largely known differences at gene expression levels, a great proportion of transcriptional perturbations occurring in breast cancer can be ascribed to differences in splicing balance. These perturbations do not necessarily affect the overall expression of genes - that is the sum of all their expressed isoforms - but relate to the balance between the different splicing variants of each gene. Large proportions attributable to splicing differences are observed in basal-like tumours when compared to all other subtypes and to NBT samples (Fig. 2). By focusing our analyses on the basal-like subtype, we identified genes and pathways that with respect to normal tissues are significantly affected by differential splicing balance. These include cancer hallmarks of various types: oncogenes (BCL2, BRAF), caspases (CASP6/7), transcription factors (E2F3), cell cycle genes (CDC42, CDK2, CDKN2A), cancer related kinases (JAK2/3, MAPK4/6/14) and DNA repair genes (BRCA1, PARP1 and RAD50).

We could also infer information on the pathways that were exclusively affected by splicing imbalances. We found a clear enrichment for pathways involving cell surface and extracellular matrix genes, controlling cellular adhesion, cellular motility and spreading. Also perturbed specifically at splicing levels are a number of key oncogenic signaling pathways mediated by cell surface receptors such as the *MAPK/ERK, mTOR and RAS* signaling pathways, as well as pathways related to the immune response. Of note, these pathways would have been completely overlooked from a gene-centric perspective (using for example Affymetrix 3′ microarrays), as they are not altered at the overall gene expression level. Relating exon level information extracted from Exon Array data to individual splicing isoforms is not straightforward and is complicated by the fact that many times the same exon can be shared across several isoforms. Despite this limitation, the general quantification of the volume of splicing imbalance events in basal-like cancers, along with the general observation that many of them involve surface proteins and oncogenic pathways, has important consequences and might open considerable translational perspectives. An example is the development of blood-based molecular assays for the detection of specific isoforms to diagnose this specific breast cancer subtype. Surface-specific cell protein isoforms are also attractive candidate therapeutic targets for development of monoclonal antibody-based therapies. This point is particularly relevant as for this subtype of breast tumours only limited therapeutic options other than systemic chemotherapy are currently available.

In the second part of our work we aimed at evaluating the potential of exon-level splicing information as biomarkers to predict clinical outcome, which represents yet another challenge posed by these tumours.

Previous demonstrations of how clinical outcome can depend on the expression of alternatively spliced isoforms in cancer suggest potential for the use of splicing information to predict patients’ prognosis. For example, RHAMM and HAS1 genes in bone marrow and TKS5 in lung have isoform imbalances that have been shown to be prognostic indicators for multiple myeloma and lung adenocarcinoma, respectively^63^,^64^,^65^.

Through parallel exon and gene level survival analyses we could disentangle associations between gene expression and exon splicing levels with clinical outcomes.

We identified 247 genes whose splicing levels were significantly associated with basal-like tumour patients’ survival, whilst the same association did not emerge from whole-gene expression analysis. Interestingly, what appears to drive the association of these genes with patients’ prognosis is the balance of different transcriptional gene isoforms rather than their overall expression levels. Among them are cancer-related genes such as MYB and TGFBR1 as well as many immune-related genes such as CCR7 and FCRL3, whose prognostic association is likely to reflect the inflammatory process and the presence of lymphocytic cells in the tumour. Indeed, when we included the percentage of lymphocytic infiltration in the model we found that the prognostic association of many of these genes is lost. Through expression analyses of lymphocytic specific genes we showed that many of these splicing variants are in fact expressed in immune cells.

These findings confirm the relevance of immune system related genes in determining tumour control or progression and extend this notion by showing that the splicing levels of many immune-related genes also hold prognostic information. Whether this is a reflection of the engagement of different T- and B-cell types in tumour inflammation, each expressing a specific isoform and with different effects on tumour progression and clinical outcome, will require further investigation.

We were also able to identify 7 genes whose splicing levels have statistically significant prognostic association, independently of the abundance of lymphocytic cells in the tumour. Among these is the TGF beta receptor TGFBR1, a membrane protein receptor involved in many cancers and whose non-synonymous single-nucleotide polymorphisms were previously observed to be associated with risk for several forms of cancer, including breast [8]. We showed that in addition to the identified SNPs TGFBR1 splicing balances also hold prognostic information.

## Conclusion

This work reveals the role of splicing mechanisms in altering key processes in basal-like breast tumours, involving cell surface proteins, immune-related and oncogenic pathways, and provides the basis for the identification of novel isoform-specific membrane therapeutic targets. Our findings disclose aspects of breast cancer transcriptional biology that have so far been largely unexplored. We investigated the use of splicing information in relation to prognosis and have identified genes whose internal splicing balances are associated with patient clinical outcome, in absence of an association at the overall gene-level of expression.

These results highlight the relevance of splicing information for translational applications as potential prognostic biomarkers and in revealing cancer specific targets for therapy. Whilst conclusive assessment of the prognostic value of each of these spliced gene exons will have to await confirmation in larger data sets, our study demonstrates the potential of splicing information as a prognostic biomarker and for the discovery of isoform-specific therapeutic targets in basal-like breast cancer.

## Materials and Methods

All methods were carried out in accordance with the approved guidelines.

### Patient characteristics and sample preparation

This study was based on the same patients’ cohort and tumour samples analyzed in previous studies by our group^13^,^14^. The clinical endpoint considered here was breast cancer specific survival (BCSS), therefore events of death due to other reasons were ignored. In addition, 9 samples of Normal breast tissue (NBT) were obtained from patients undergoing mammoplasty for aesthetic reasons, under protocols approved by the Institutional Review Board and by Guy’s Research Ethics Committee, in compliance with the Human Tissue Act. Informed consent was obtained from all subjects from where the tissue samples were taken. The tissues were processed as described in ref. 66. Exon-level transcriptional profiles were obtained by using the Affymetrix Exon 1.0 ST array platform. Tumour molecular subtypes were assigned as described in ref. 13.

### Exon-Array data pre-processing

An overview of the workflow used for Exon-Array data pre-processing is given in Additional File 19 following the analytical strategy proposed by Lockstone *et al*.^67^. ExonArray data pre-processing was performed on the R platform using the “aroma.affymetrix” R package (www.aroma-project.org). RMA was used to remove the array signal background, followed by quantile normalisation to correct for inter-arrays global differences and by gene level summarisation. For this latter step probe sets were mapped to ENSEMBL genes using the mapping file (HuEx-1_0-st-v2, U-Ensembl49, G-Affy.cdf) generated by the aroma.affymetrix team^68^. Quality of individual arrays was assessed by visual evaluation of RLE (relative log expression), NUSE (normalised unscaled standard error) and hierarchical clustering plots (Additional File 19).

Once expression levels were obtained for each gene and probe set, they were tested for differential expression between different sample groups. Tumour samples were annotated according to the PAM50 molecular classification and on the basis of the ER, PR and HER2 status^1^. Gene level expression measures were tested for differential expression using the moderated t-test implemented in the Limma package (http://www.bioconductor.org/packages/release/bioc/html/limma.html) as part of the R/Bioconductor platform^69^. Likewise, for the exon analysis, the expression recorded for each probe set was evaluated and compared in the same way. With the genomic mapping of probe sets coordinates of the hg19 genome assembly, they can be mapped on to specific gene and exon locations. The obtained p values were corrected for multiple hypotheses testing using the Benjamini and Hochberg method^70^ and the resulting corrected values are hereafter referred to as q values. Except where otherwise noted, a gene was considered to be differentially expressed when its q value for the test is lower than 0.001.

Splicing Index (SI) values were calculated by dividing expression values captured for each probe by the sum of the expression values of all the probes of that gene, as reported elsewhere^19^. Differential splicing index between samples was then tested with the identical procedure: *i.e.* using the SI as input values to the Limma package to calculate p values of as described above. By using the SI metric it is possible that when comparing two groups of samples, the average SI of one exon is higher (e.g. due to exon inclusion or to higher expression of an isoform containing that exon), and the average SIs for the other exons of the same gene are lower. In this case our analysis would detect an overall splicing imbalance for that gene, due to different SI values of the exons in the two samples. Splicing imbalances are reported at the gene level; therefore results are not affected if the imbalance is detected from one or more exons within the same gene. The p-values were adjusted for multiple hypotheses testing using the Benjamini Hochberg method^70^ and the resulting corrected values are referred to as q values. A gene was deemed to have a splicing imbalance between two groups when the q value in one or more of its probes was lower than 0.001.

The comparison of multiple pairwise combinations of subtypes needs also to be taken into account as a further element for multiple testing correction. However, standard multiple testing procedures assume independence of individual tests and our pairwise comparisons violate the assumption of independence (i.e. subtypes of the same cancer type cannot be considered independent). We have addressed this problem by using a very stringent threshold (q-val < 0.001), which accounts also for the multiple pairwise subtypes comparisons (n. of tests = 10).

### Analysis of p-value distributions from pairwise comparisons

Distribution of p-values obtained for each pairwise comparison were compared against the theoretical distribution under the null hypothesis of differential expression as the result of random noise. The latter was modeled in two ways: (i) as the uniform distribution (ii) as the Montecarlo distribution obtained upon permutation of sample labels. In all cases distribution of p-values obtained for pairwise comparisons showed clear deviation from the null-hypothesis distribution(s), indicating the presence of statistically significant signals in the data.

### Overlaps with external data sets

Lists of genes with differential splicing balances were extracted from our data upon pairwise comparisons between TNBC or basal-like tumour samples with luminal tumours or NBT samples. These lists were compared with equivalent lists published in refs 8, 9 as described in the results section. A third comparison was done against RNA-Seq data downloaded from The Cancer Genome Atlas project (TCGA, http://cancergenome.nih.gov/). The data was in the form of “Level 3 data” according to the TCGA nomenclature, which represents gene and isoform level read counts. All samples for which the status of ER, PR and HER2 receptors was available, annotated as “basal-like” according to the PAM50 molecular classification^1^ were used in the subsequent analysis. Differential gene expression and differential isoform expression between basal-like tumour (n = 92) and NBT samples (n = 133) was calculated using edgeR^71^. Genes and isoforms with q-value lower than 0.001 were considered to be differentially expressed. From that data we compiled a list of genes having at least an isoform differentially expressed, but not found to be differentially expressed when analyzed at overall gene-level. Similarly, from our data, we selected genes which had one probe indicating differential splicing index (q-value < 0.001) but not found to be differentially expressed when analyzed at overall gene-level. Considering as background population of genes all the gene symbols that could be mapped to isoform names and Ensemble gene Ids, we calculated the statistical significance of the overlap of these two gene lists using the hypergeometric test.

### Experimental validation of differential splicing results

We have selected 11 genes in total, to be either differentially spliced between basal-like tumours and normal samples (8 genes: AURKA, AURKB, BCL2-a, NEK2, RRM2, TGFBR1, UBE2C, ZBTB16) or differentially spliced in basal-like tumours, between patients with respectively better and worse outcome (4 genes: CCR7, RASSF5, PARP12, TGFBR1). Full length cDNAs were prepared from intact RNA using Primescript Reverse transcriptase (Clontech), oligo dT and a custom transcript switching Oligo (TSO). cDNAs were amplified using semi-nested PCR using the Advantage PCR kit (Clontech) with gene specific primers located near the poly adenylation signal and TSO. Amplified full length cDNAs were analysed using Bioanalyzer DNA7500 kit to size separate all full length isoforms arising from each gene (Additional File 8).

### Analysis of cellular functions and pathways

We evaluated what cellular functions and pathways were affected at the gene splicing balance or gene expression levels. We analyzed separately a number of gene lists derived from comparative analyses of differential splicing index (splicing imbalance) and differential gene expression, using gene set enrichment analyses based on Fisher-test. The gene sets we used were extracted from the Ingenuity (www.ingenuity.com) as well as the MSigDB data base^72^.

Ingenuity gene sets were used for the analysis of the list of genes differentially spliced (therefore had differential splicing index values) but not differentially expressed, between basal-like tumours and breast normal tissues. MSigDB was used for all comparative lists. For any gene list the Fisher-test assessed the probability that the number of overlapping genes between our gene list and the pre compiled gene set would happen by chance. The background population for the test consisted of all genes represented on the Affymetrix GeneChip Exon 1.0 ST platform.

### Classification models

Classification models were built to assess the diagnostic potential of three different levels of information that can be extracted from Exon Array: gene expression, exon expression, and splicing index. An additional data type was used consisting of all splicing indexes of the exons of genes not differentially expressed between the categories to be classified (*i.e.* the q value for difference in the expression of that gene was greater than 0.01). The classification model was, in all cases, based on decision trees as implemented in the R package “tree” (http://cran.r-project.org/web/packages/tree/index.html). For each data type the procedure used was the same: (1) *n* number of variables were selected randomly from the data matrix (for example gene expression values) (2) we randomly assigned two thirds of biological samples to be the *training samples*. Those are used to calibrate the model using the *n* variables. (3) the model is then used to classify the remaining third of samples (the *test samples*). For every number *n* of variables this procedure (steps 1–3) is repeated 1000 iterations always randomly selecting the test and training samples, as well as the specific *n* variables to use. From those classifications we calculated sensitivity and selectivity associated to each model.

### Survival Analysis

Kaplan-Meier analysis was used for calculation and visualization of survival curves, and Cox-regression models followed by Wald test were used to determine the statistical association between the expression of each GE, EE, SI value and breast cancer specific survival (BCSS). Two different Cox-regression models were used, with or without consideration of the percentage of lymphocytic infiltration as an additional covariate. To adjust for multiple testing, false discovery rate (FDR) q-values were calculated from the Wald test p-values, using Benjamin-Hochberg method. We considered GE, EE, SI to be associated with BCSS using FDR q-value < 0.1. Distributions of the resulting p-values were compared with random uniform distribution (from 0 to1), representing p-values that would be obtained by chance. With all models the obtained p-values were clearly deviating from uniform distribution with an overall bias towards low p-values, and therefore deviating from the results that would be obtained by chance (Additional File 12). Percentage of lymphocytic infiltration covariate was used as a categorical variable, as follows: <15% = “low”, >=15% = “high”. All analyses were run using R software and the ‘survival’ R package (http://cran.r-project.org/web/packages/survival/index.html).

### Annotation of Lymphocytic associated Genes

Genes were annotated as lymphocytic according to two criteria: i) expression in lymph node tissues, based on the arbitrary threshold of at least 10 read counts from the normalized RNA-Seq data set deposited in the Array Express database, data set E-MTAB-513 (http://www.ebi.ac.uk/gxa/experiments/E-MTAB-513) ii) the gene was present in any of the following data sets from the MSigDb^72^: “BIOCARTA_BLYMPHOCYTE_PATHWAY”,“REACTOME_IMMUNOREGULATORY_INTERACTIONS_BETWEEN_A_LYMPHOID_AND_A_NON_LYMPHOID_CELL”,“SIG_PIP3_SIGNALING_IN_B_LYMPHOCYTES”.“PID_LYMPHANGIOGENESIS_PATHWAY”,“LYMPHOCYTE_DIFFERENTIATION”,“POSITIVE_REGULATION_OF_LYMPHOCYTE_ACTIVATION”,“REGULATION_OF_LYMPHOCYTE_ACTIVATION”, “LYMPHOCYTE_ACTIVATION”.

### Availability of supporting data

Patient clinical and pathological information used for the analyses, are reported in Table S1 and include the patients’ survival data, age at diagnosis, tumour grade, percentage of lymphocytic infiltration, estrogen (ER), progesterone (PR) and human epidermal growth factor receptor 2 (HER2) status.

Microarray data have been deposited in GEO public repository with ID GSE40267 (http://www.ncbi.nlm.nih.gov/geo/query/acc.cgi?acc=GSE40267).

## Additional Information

**How to cite this article**: Gracio, F. *et al*. Splicing imbalances in basal-like breast cancer underpin perturbation of cell surface and oncogenic pathways and are associated with patients’ survival. *Sci. Rep.* **7**, 40177; doi: 10.1038/srep40177 (2017).

**Publisher's note:** Springer Nature remains neutral with regard to jurisdictional claims in published maps and institutional affiliations.

## Supplementary Material

Supplementary Information

Supplementary Dataset 1

Supplementary Dataset 7

Supplementary Dataset 9

Supplementary Dataset 14

Supplementary Dataset 20
