# Supplementary material for: Splicing imbalances in basal-like breast cancer underpin perturbation of cell surface and oncogenic pathways and are associated with patients’ survival
Source: Sci Rep. 2017 Jan 6;7:40177. doi: 10.1038/srep40177 (PMC5216415; doi:10.1038/srep40177)
Supplement: Supplementary Information [file srep40177-s1.pdf]

Splicing imbalances in basal-like breast cancer underpin perturbation of cell surface and oncogenic pathways and are associated with patients' survival

Filipe Gracio, Brian Burford, Patrycja Gazinska, Anca Mera, Aisyah Mohd Noor, Pierfrancesco Marra, Cheryl Gillett, Anita Grigoriadis, Sarah Pinder, Andrew Tutt* and Emanuele de Rinaldis*

*corresponding authors

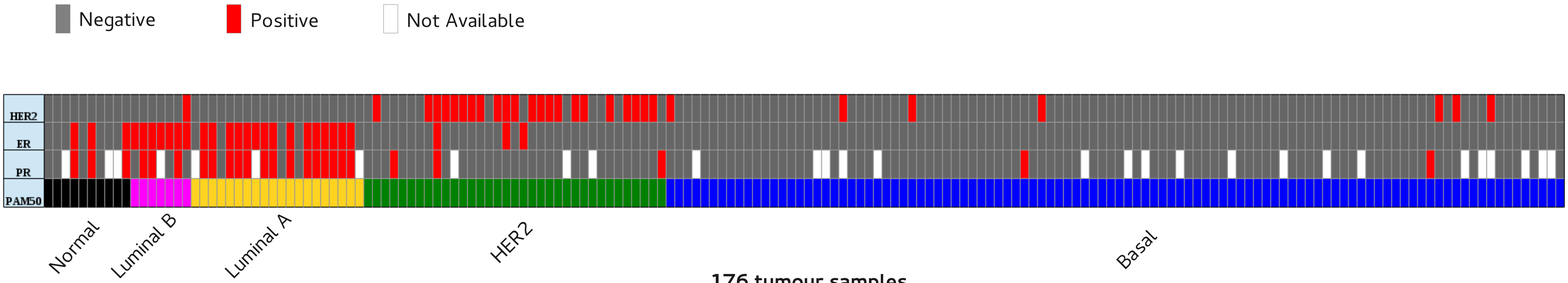

176 tumour samples

IHC–based Classification	ER–	N=148 (84%)
	ER+	N=28 (16%)
	HER2+	N=30 (17%)
	ER– PR– HER2– (Triple–Negative)	N=93 (53%)
Molecular Subtype	Basal–Like	N=104 (59%)
	HER2	N=35 (20%)
	Luminal A	N=20 (11%)
	Luminal B	N=7 (4%)
	Normal–like	N=10 (6%)

Supplemental File 2

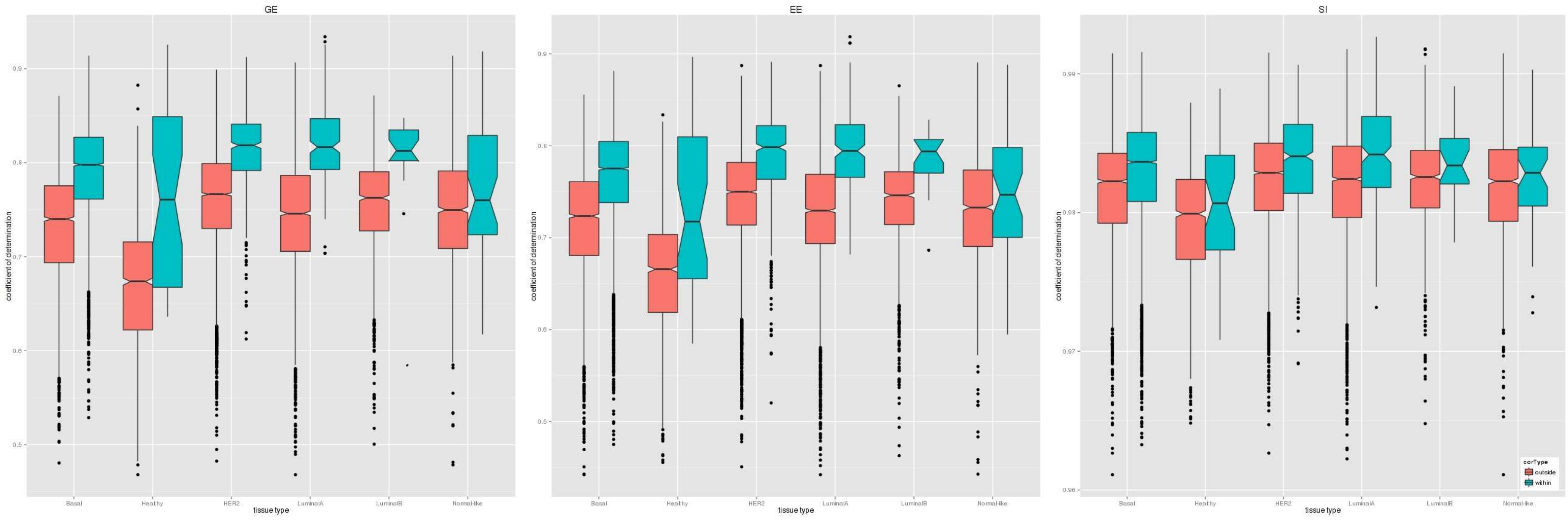

Supplemental File 3

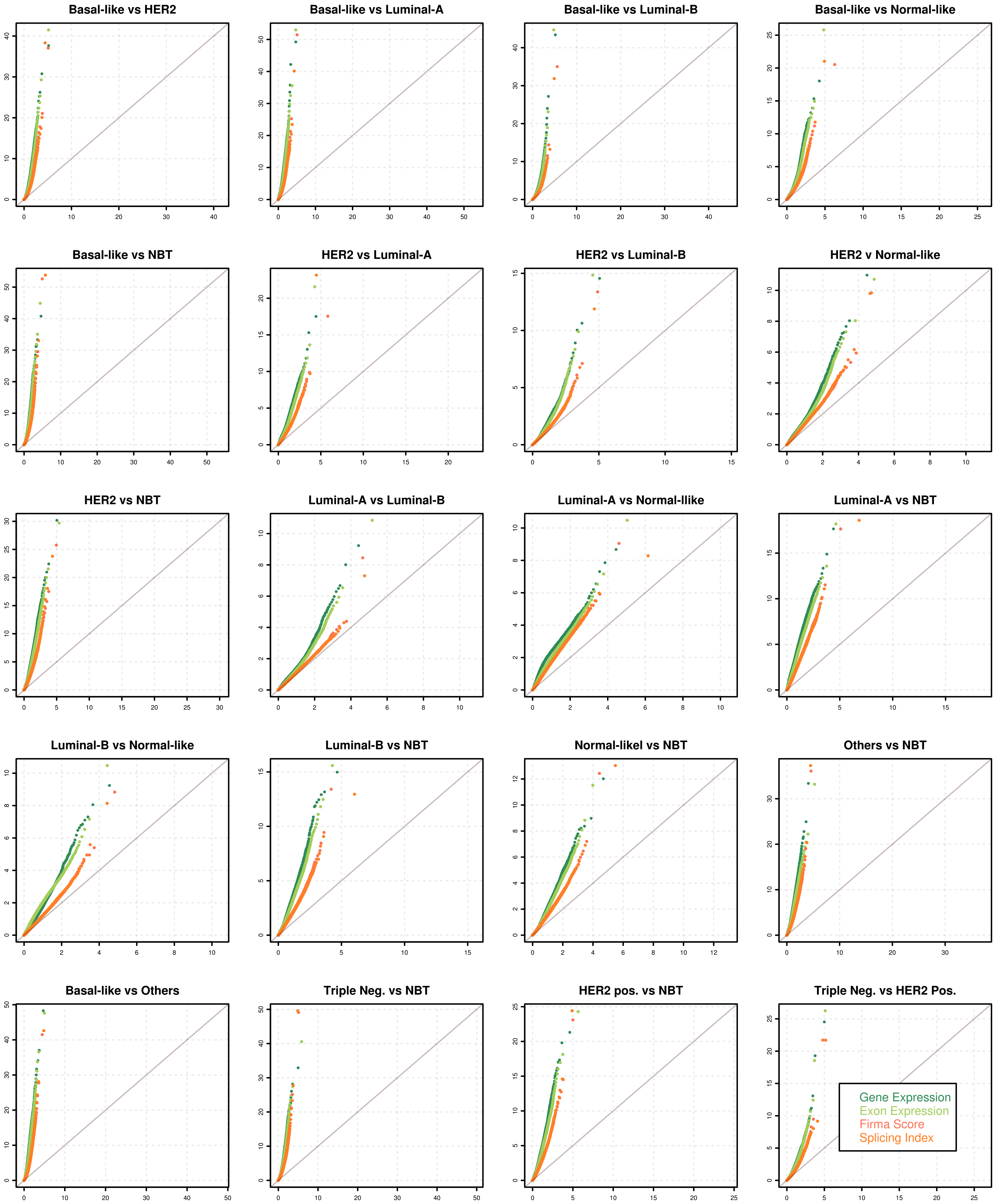
Basal-like vs HER2
Basal-like vs Luminal-A
Basal-like vs Luminal-B
Basal-like vs Normal-like
Basal-like vs NBT
HER2 vs Luminal-A
HER2 vs Luminal-B
HER2 v Normal-like
HER2 vs NBT
Luminal-A vs Luminal-B
Luminal-A vs Normal-llike
Luminal-A vs NBT
Luminal-B vs Normal-like
Luminal-B vs NBT
Normal-likel vs NBT
Others vs NBT
Basal-like vs Others
Triple Neg. vs NBT
HER2 pos. vs NBT
Triple Neg. vs HER2 Pos.
Gene Expression
Exon Expression
Firma Score
Splicing Index

Supplemental File 4

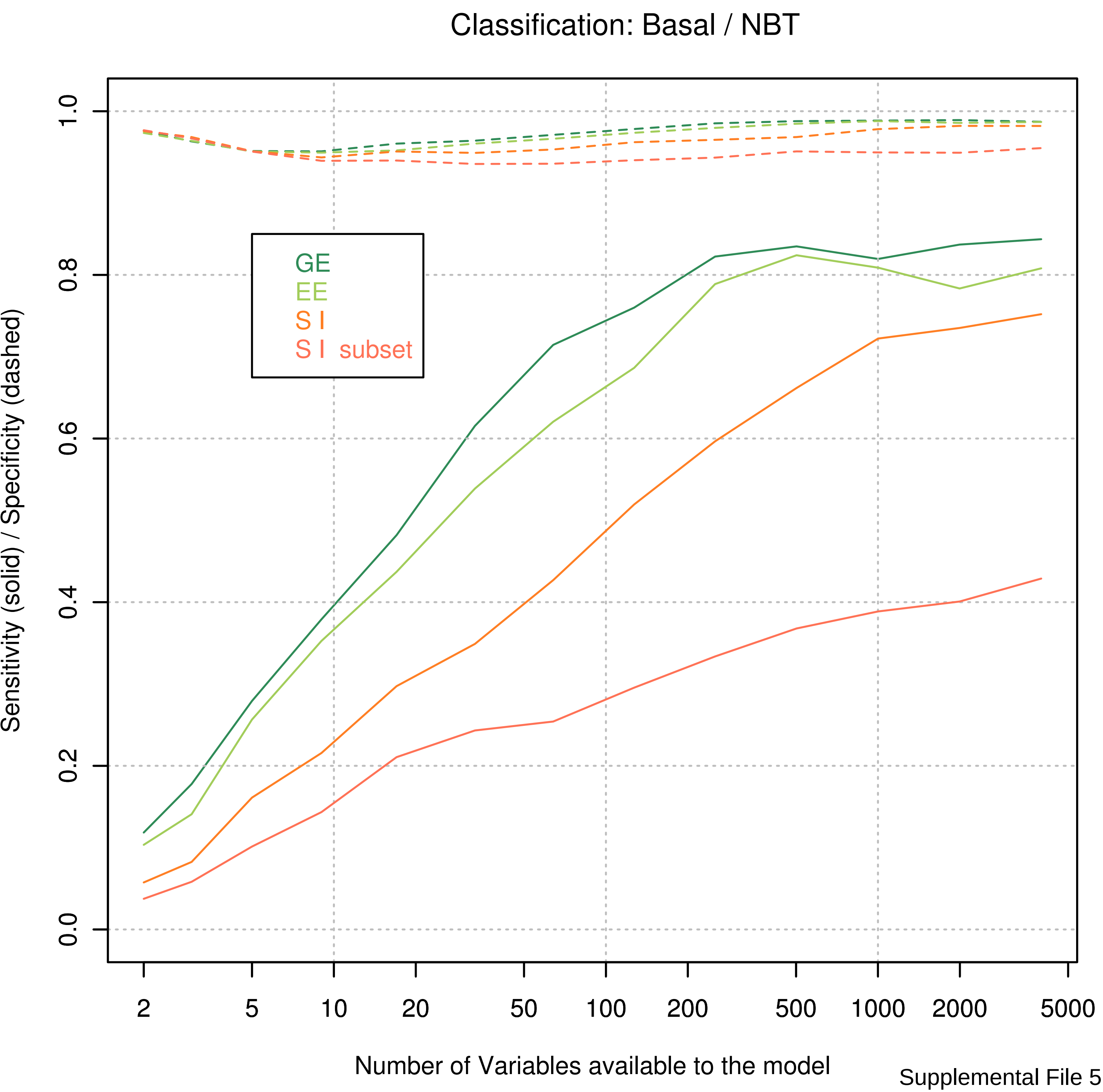

Supplemental File 5

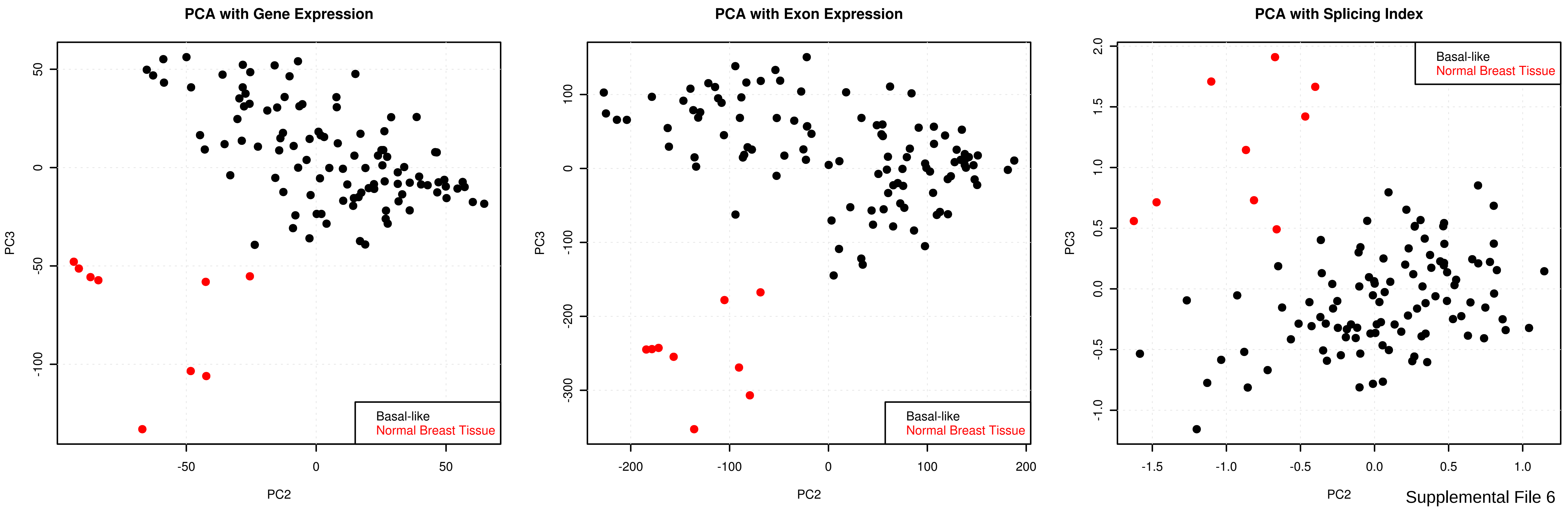

Supplemental File 6

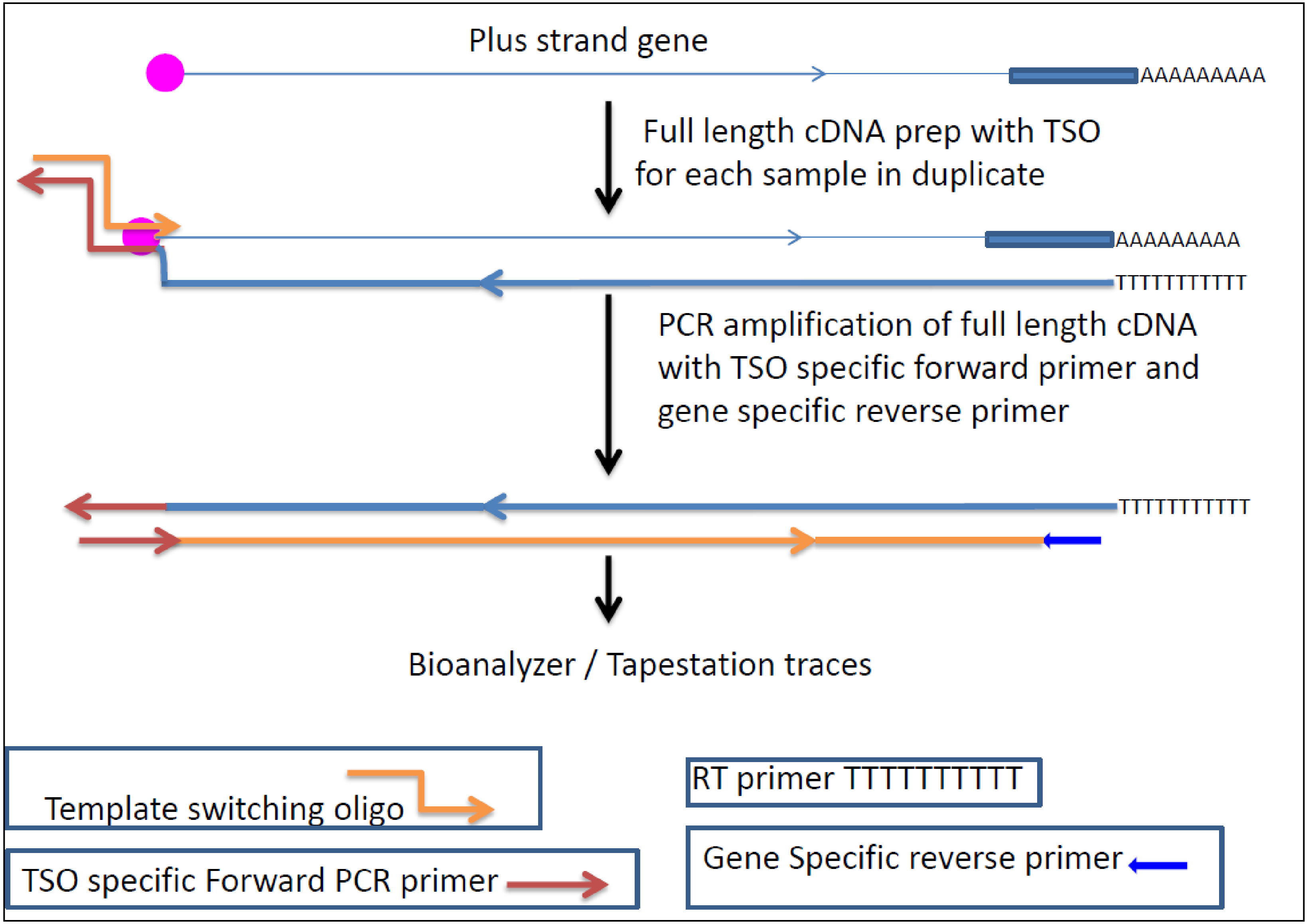

Experimental protocol for generation of amplified full length cDNAs for all RNA isoforms of a given gene

Supplemental File 8

AURKA

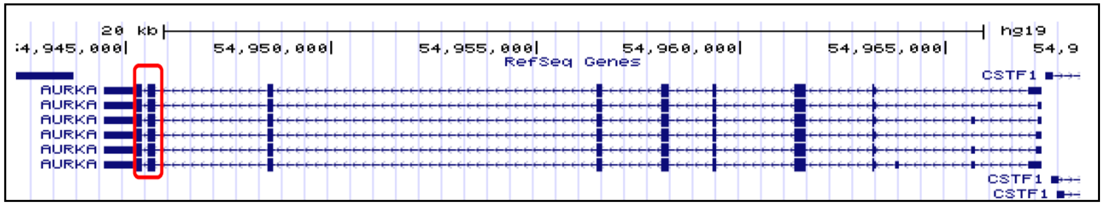

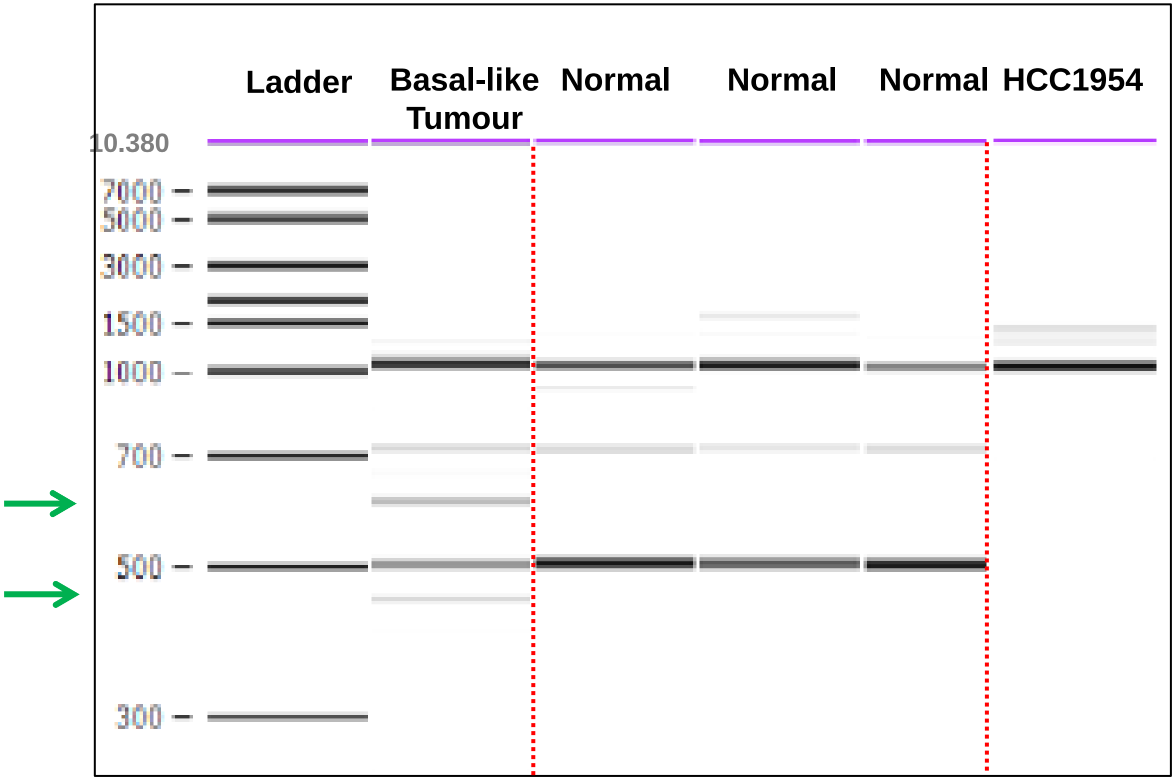

Gel-like pseudo-image obtained from Bioanalyzer analysis of amplified isoforms. Green arrows highlight isoforms differential expressed between basal-like tumours and normal samples

AURKB

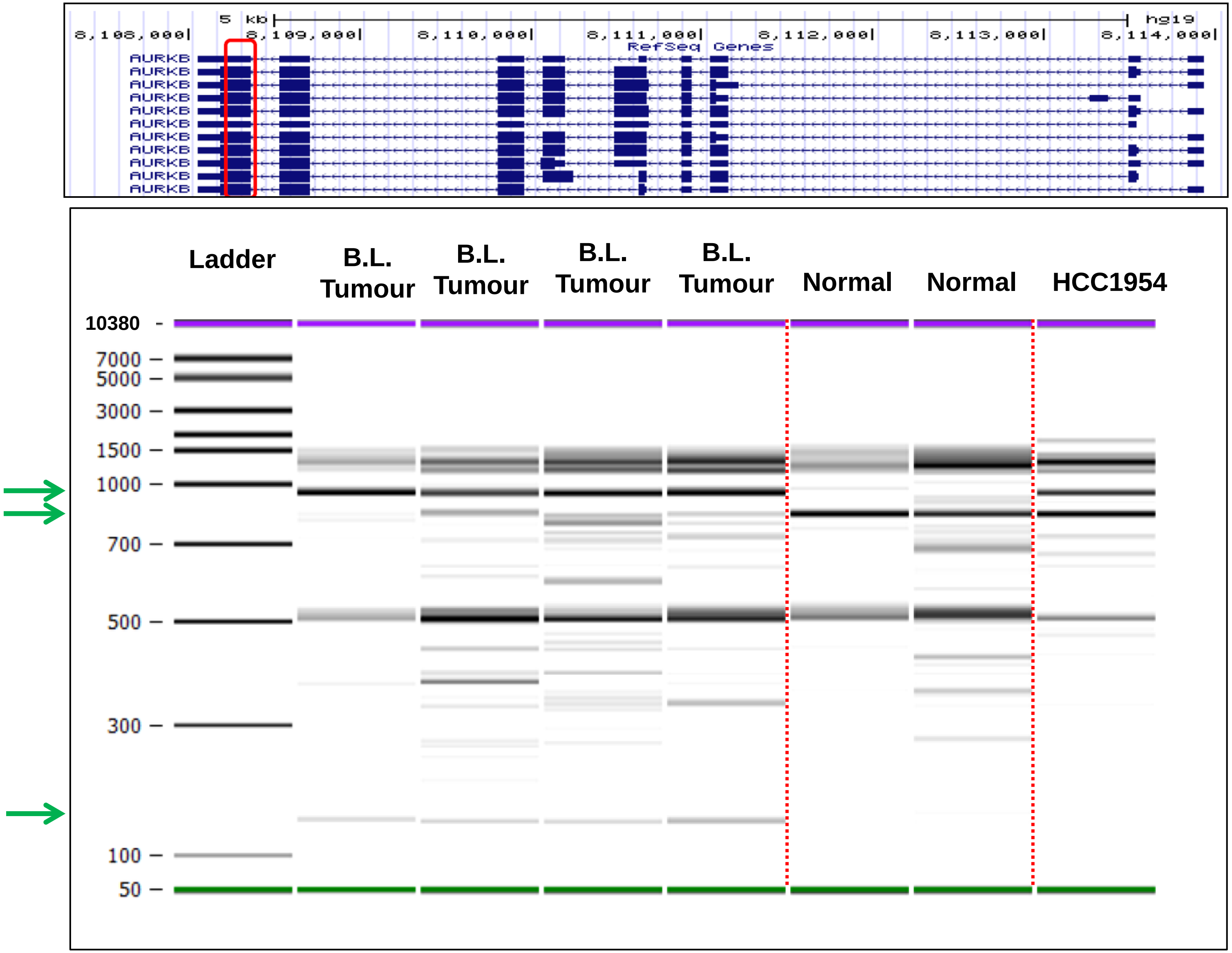

Gel-like pseudo-image obtained from Bioanalyzer analysis of amplified isoforms. Green arrows highlight isoforms differential expressed between basal-like tumours and normal samples

BCL2-α

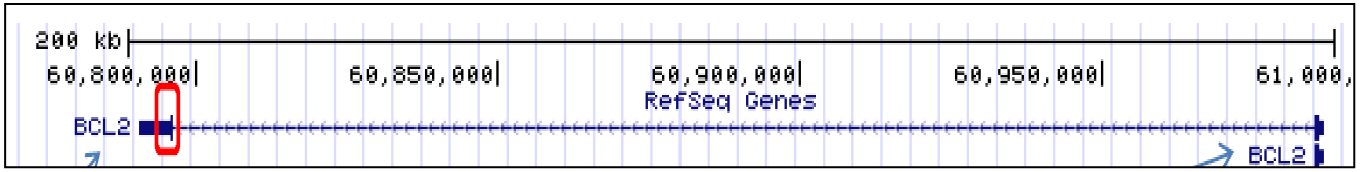

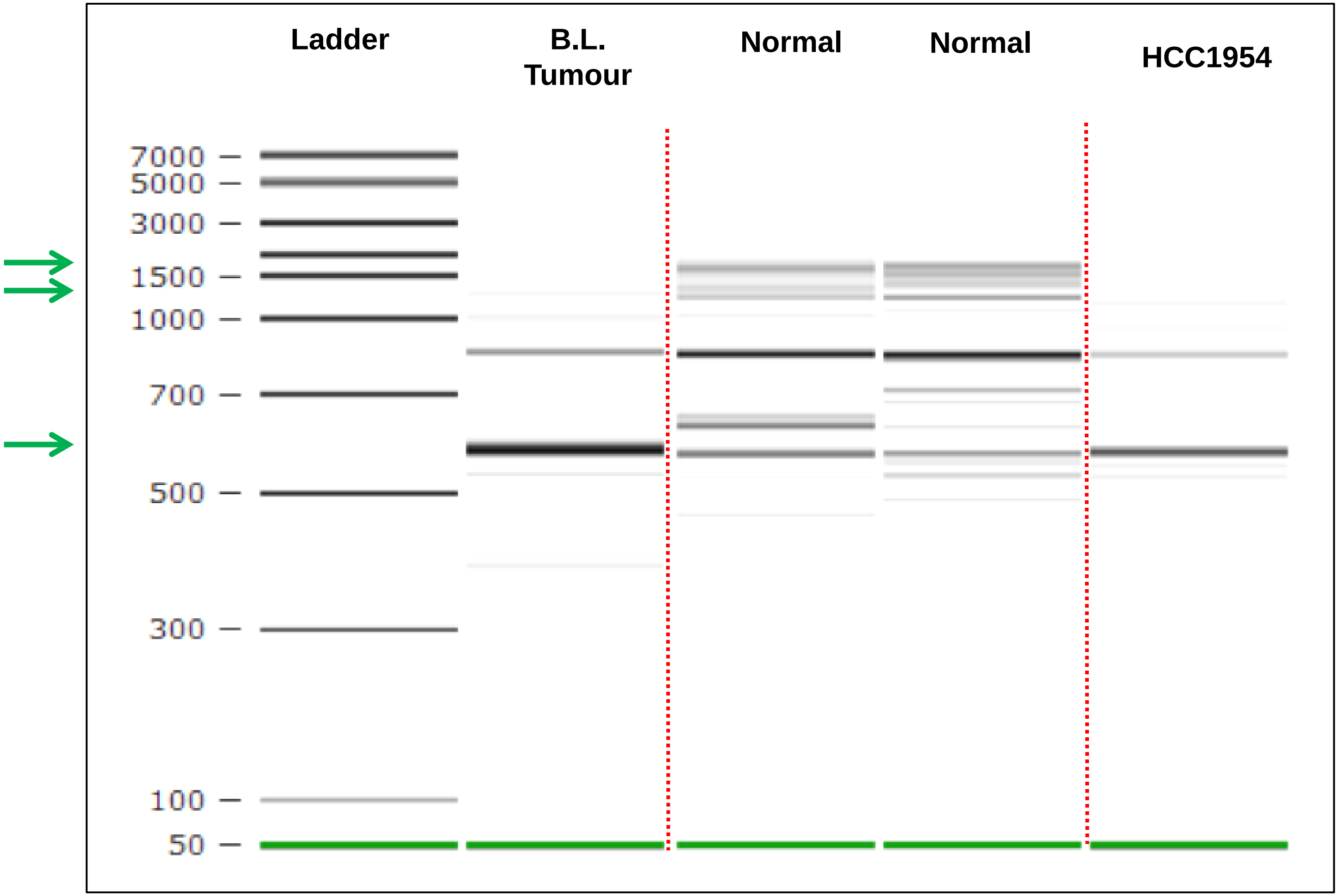

Gel-like pseudo-image obtained from Bioanalyzer analysis of amplified isoforms. Green arrows highlight isoforms differential expressed between basal-like tumours and normal samples

NEK2

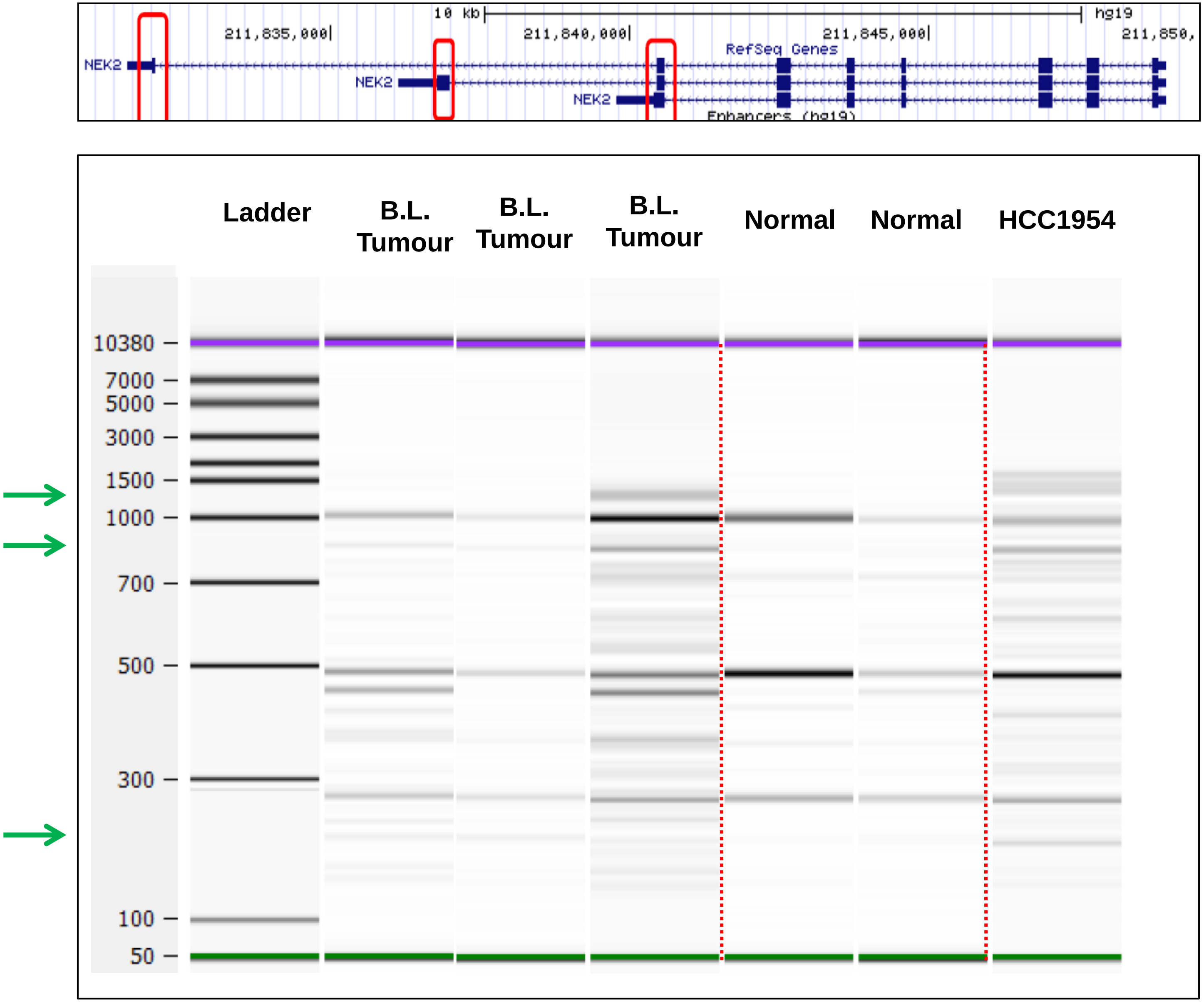

Gel-like pseudo-image obtained from Bioanalyzer analysis of amplified isoforms. Green arrows highlight isoforms differential expressed between basal-like tumours and normal samples

RRM2

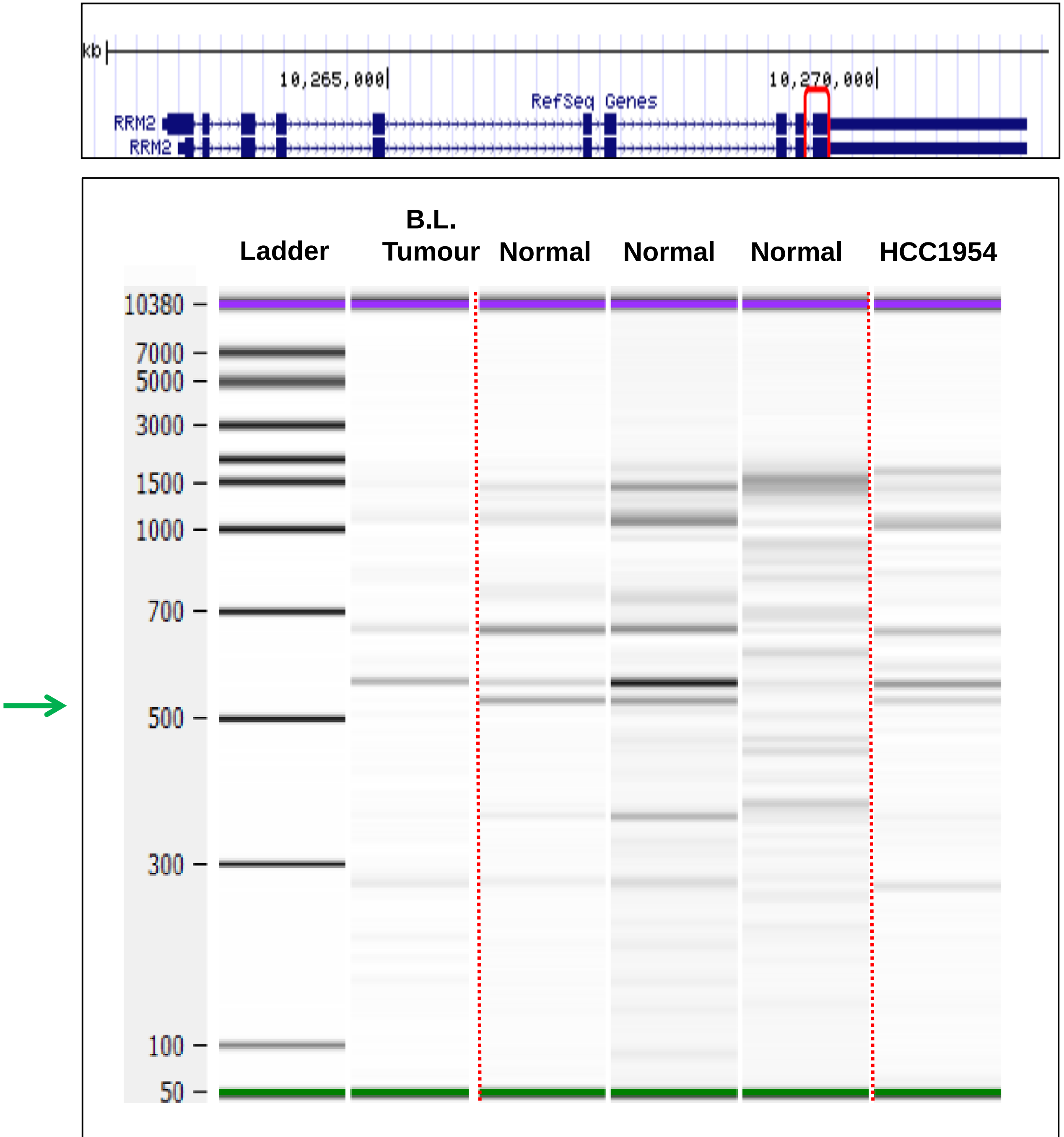

Gel-like pseudo-image obtained from Bioanalyzer analysis of amplified isoforms. Green arrows highlight isoforms differential expressed between basal-like tumours and normal samples

TGFBR1

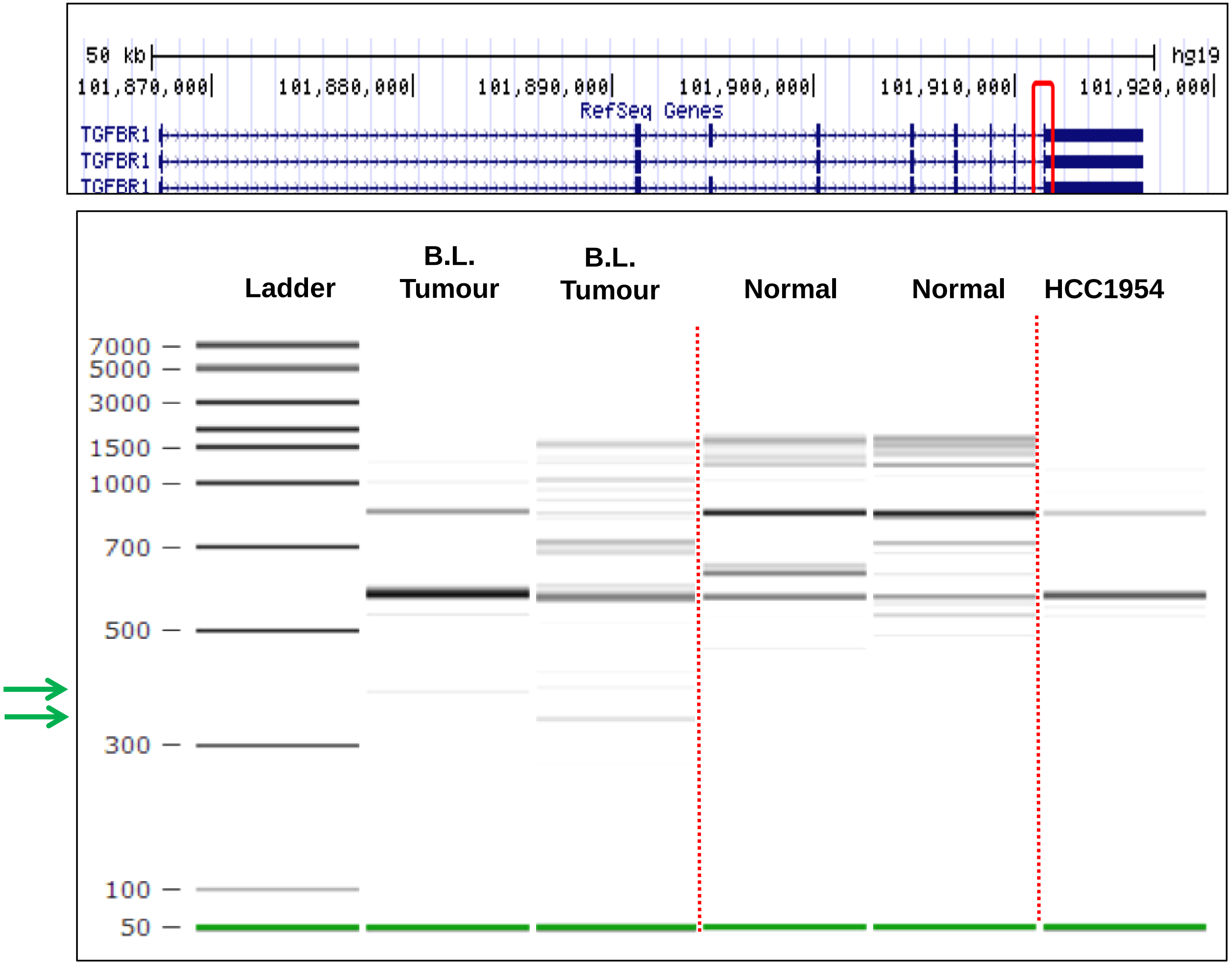

Gel-like pseudo-image obtained from Bioanalyzer analysis of amplified isoforms. Green arrows highlight isoforms differential expressed between basal-like tumours and normal samples

UBE2C

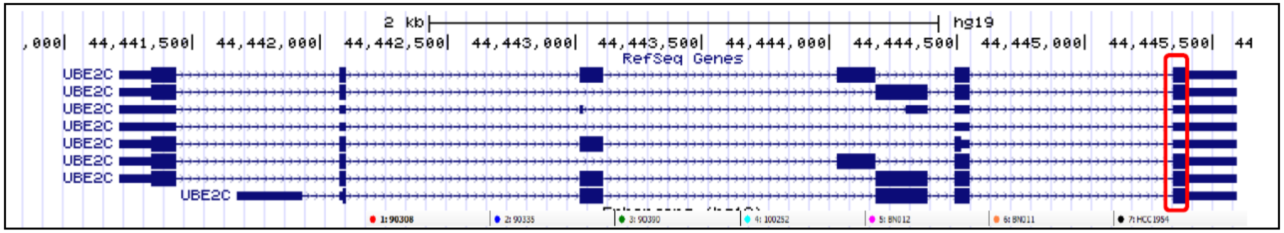

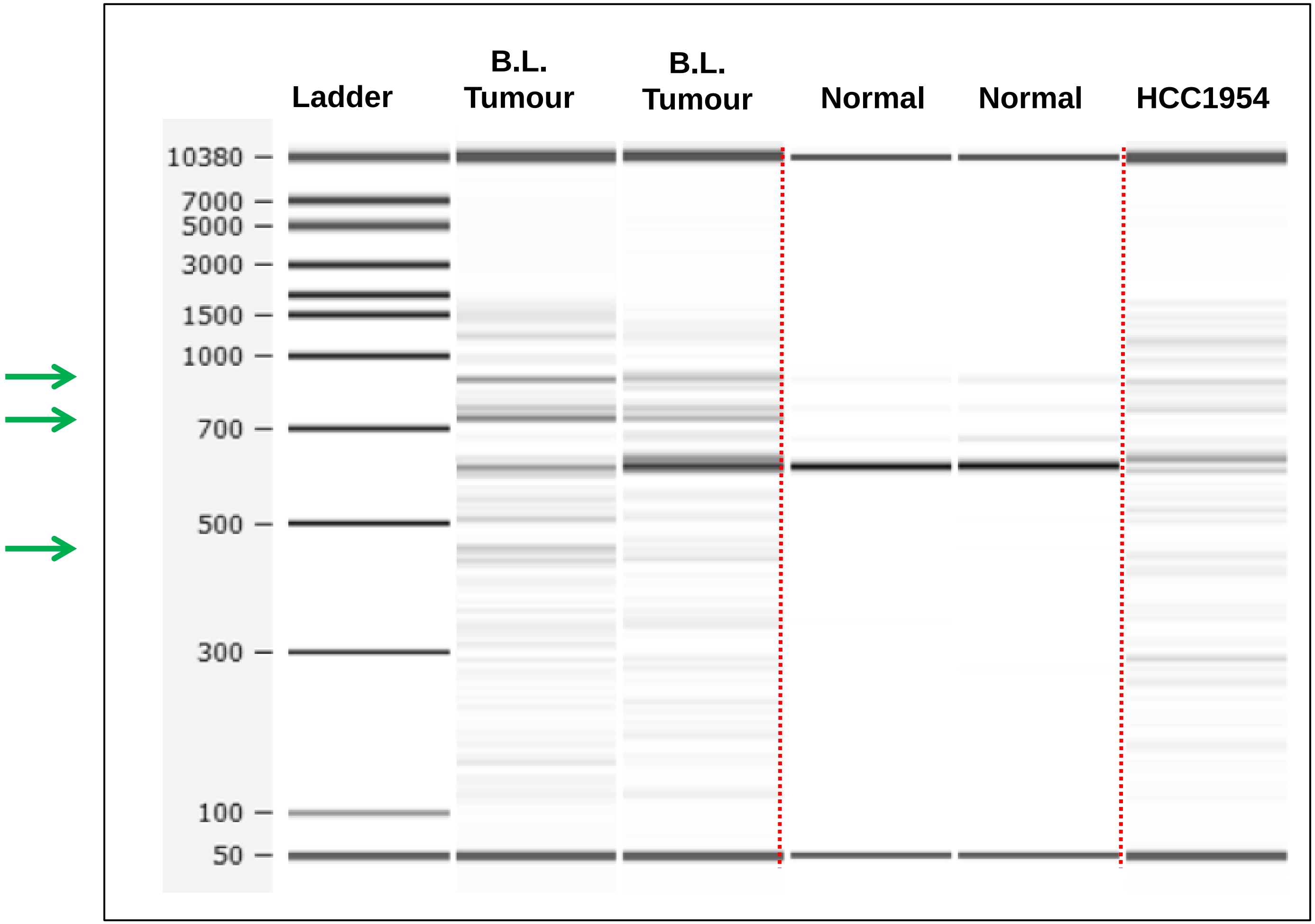

Gel-like pseudo-image obtained from Bioanalyzer analysis of amplified isoforms. Green arrows highlight isoforms differential expressed between basal-like tumours and normal samples

ZBTB16

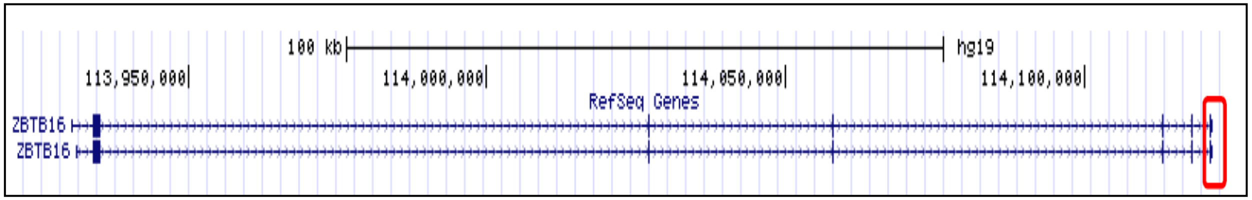

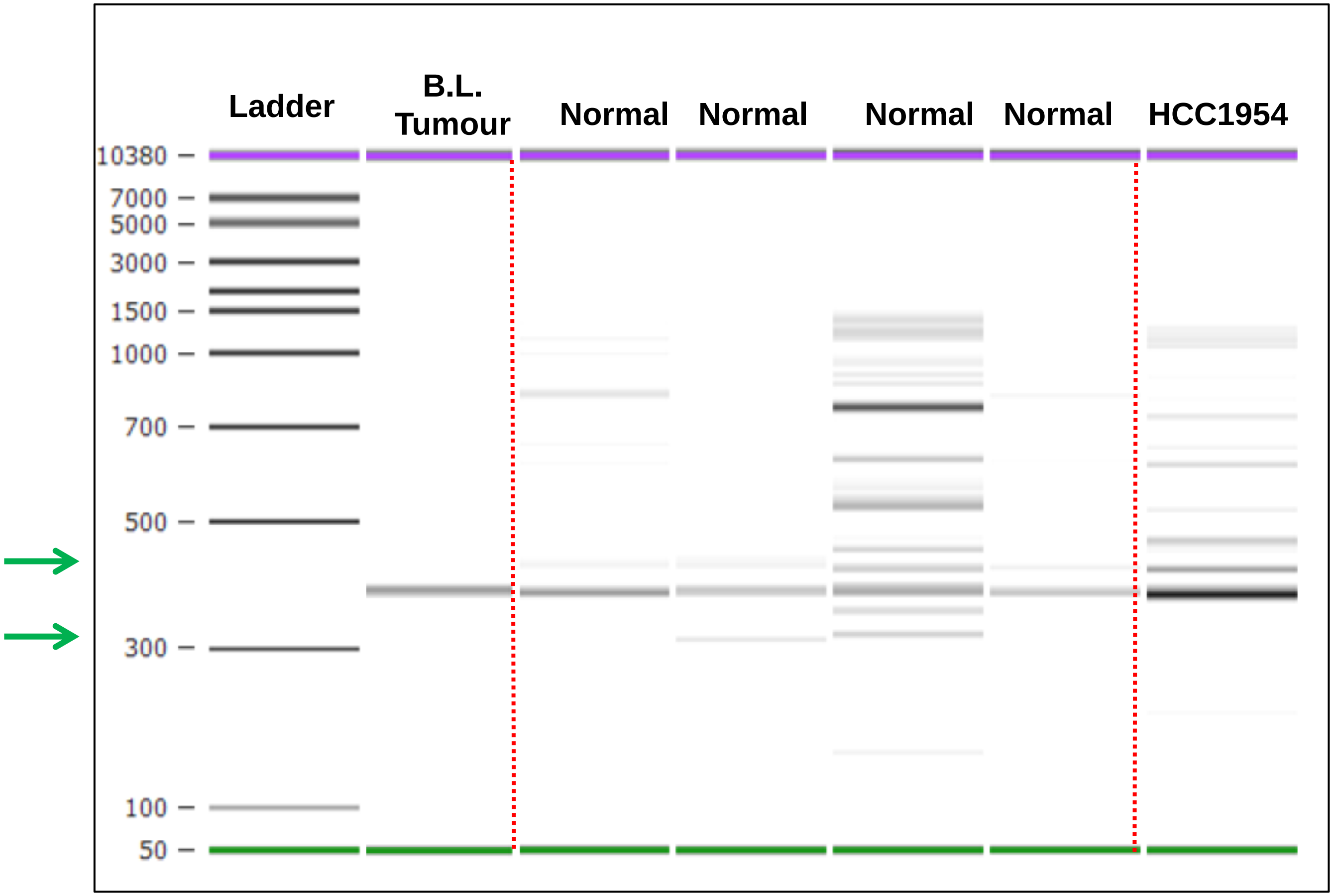

Gel-like pseudo-image obtained from Bioanalyzer analysis of amplified isoforms. Green arrows highlight isoforms differential expressed between basal-like tumours and normal samples

CCR7

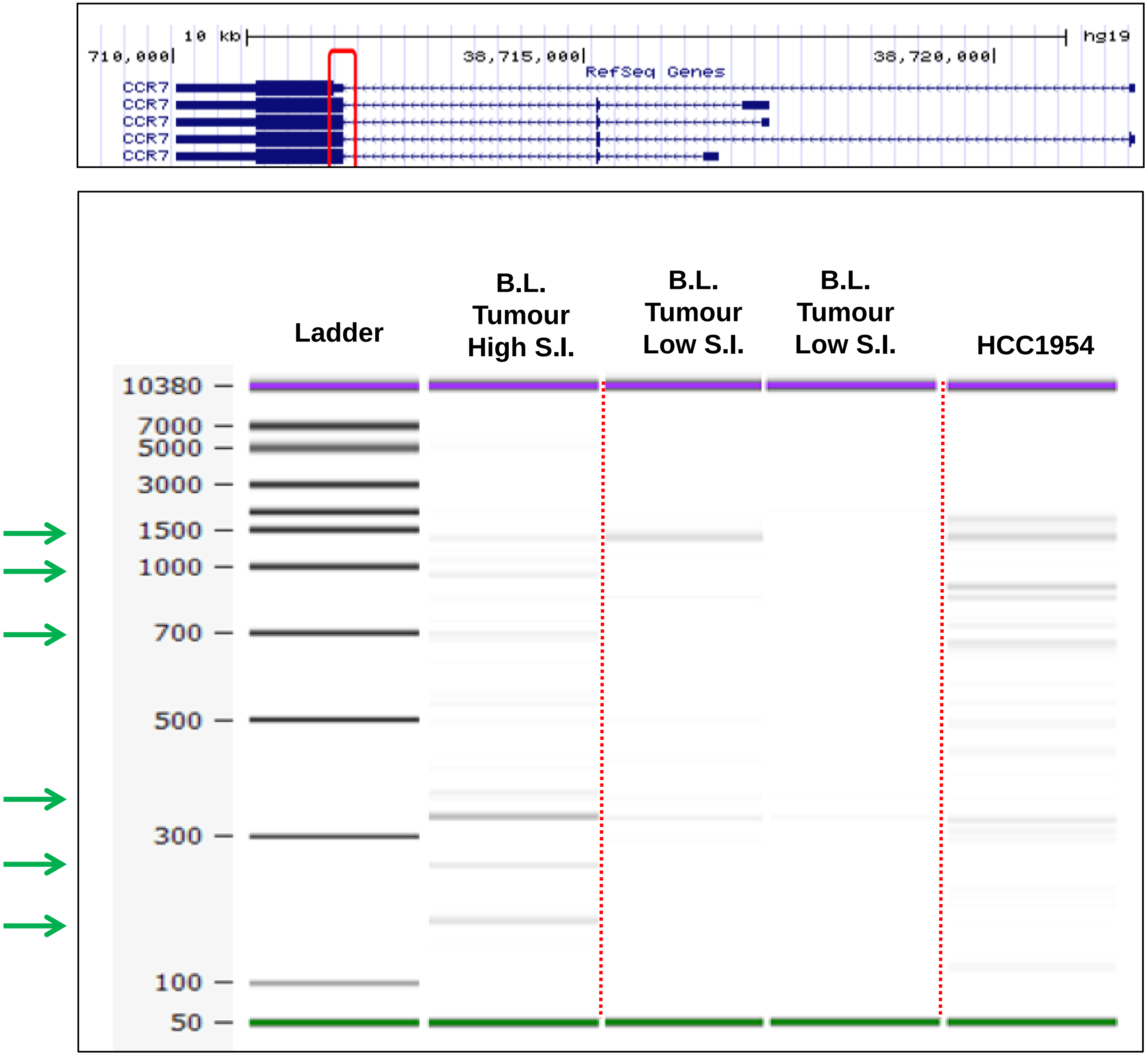

Gel-like pseudo-image obtained from Bioanalyzer analysis of amplified isoforms. Green arrows highlight isoforms differential expressed between basal-like tumours with respectively high and low splicing index

Paxillin Signaling

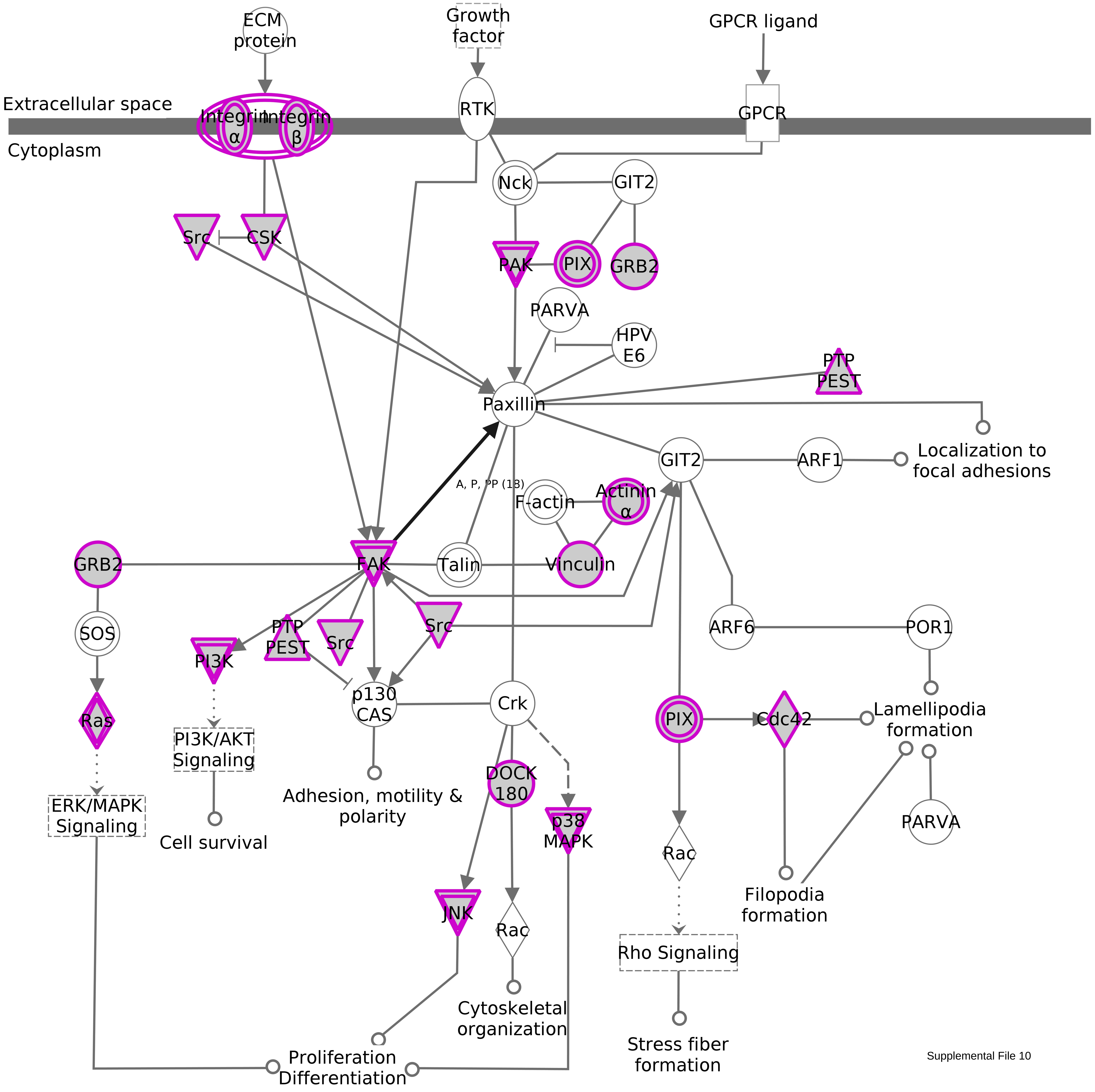

Supplemental File 10

© 2000-2013 Ingenuity Systems, Inc. All rights reserved.

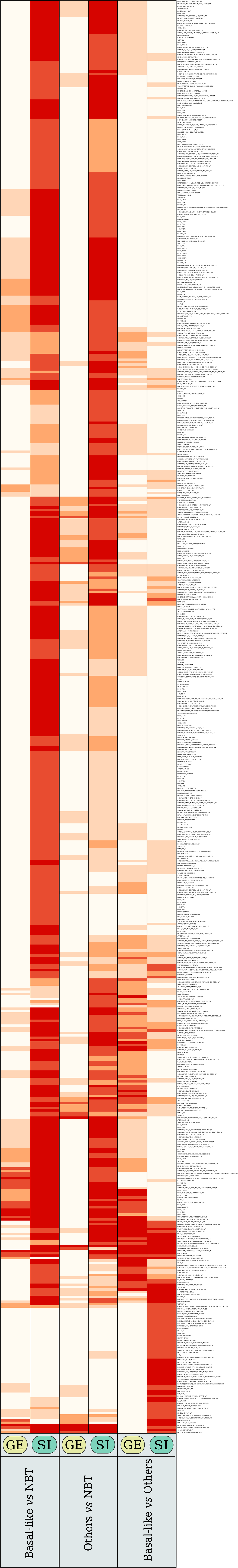
GE
SI
GE
SI
GE
SI
Basal-like vs NBT
Others vs NBT
Basal-like vs Others

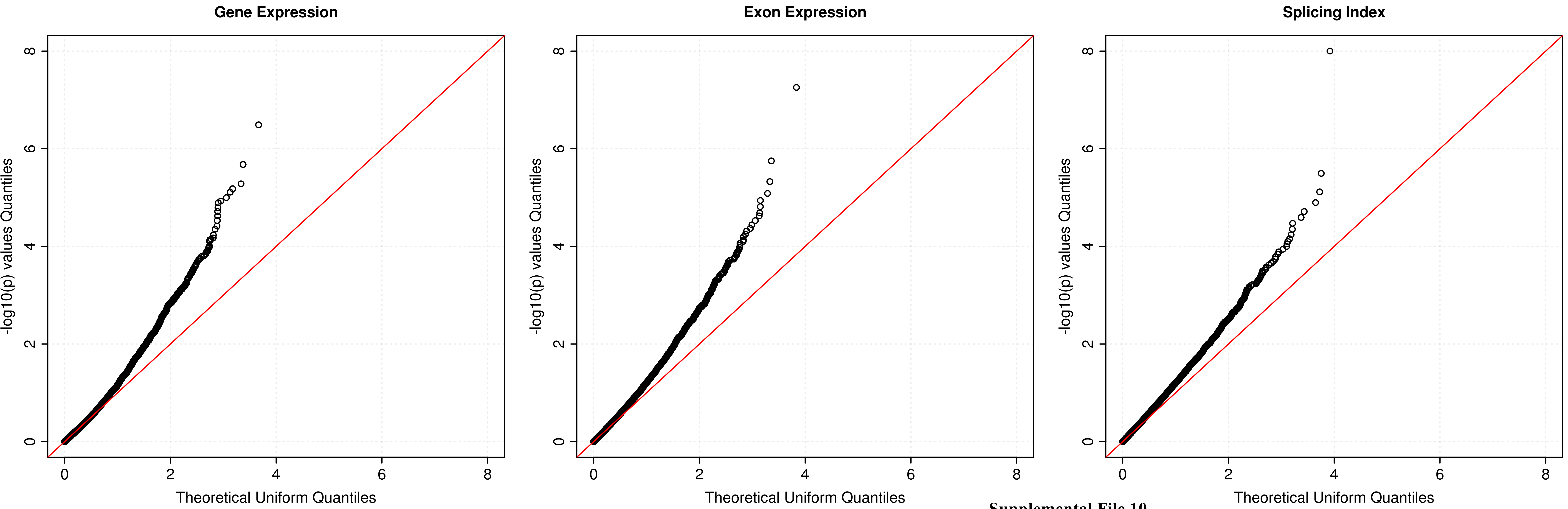

Supplemental File 10

Supplemental File 13

Validation of survival analysis results using external data sets

To our knowledge, the only external dataset publically available with exon level results along with clinical outcome is the RNA-Seq dataset deposited in Tumor Cancer Genome Atlas (TCGA) (http://cancergenome.nih.gov/). This dataset encompasses 921 breast cancer samples annotated according with PAM50 subtype classification, of which 140 are annotated as basal-like,

Our plan was to first check concordance at gene-level, and then to proceed with the further validation comparison at exon- and splicing-index level.

We tried to validate our gene-level results by comparing genes whose overall expression was associated with prognosis in basal-like breast cancer in both data sets. We took prognostic genes in basal-like tumours from our data set (204 genes having q-val < 0.1) and compared them with an equivalent number of genes with lowest p-values for association to prognosis from the TCGA data set (to be noticed that none of the TCGA genes passed the threshold of q-value < 0.1). Surprisingly, none of our prognostic genes was found in the list of TCGA genes.

To understand these results, we then explored further the TCGA dataset and run survival analyses across all PAM50 subtypes. As a result, we found that in this data set the basal-like subtype does not show up as the most aggressive subtype, in overt contrast with all previous results published on the same subject [1-3]. Even more puzzling was the observation that he subtype with the worst prognosis is the Normal-like one (see Figure below).

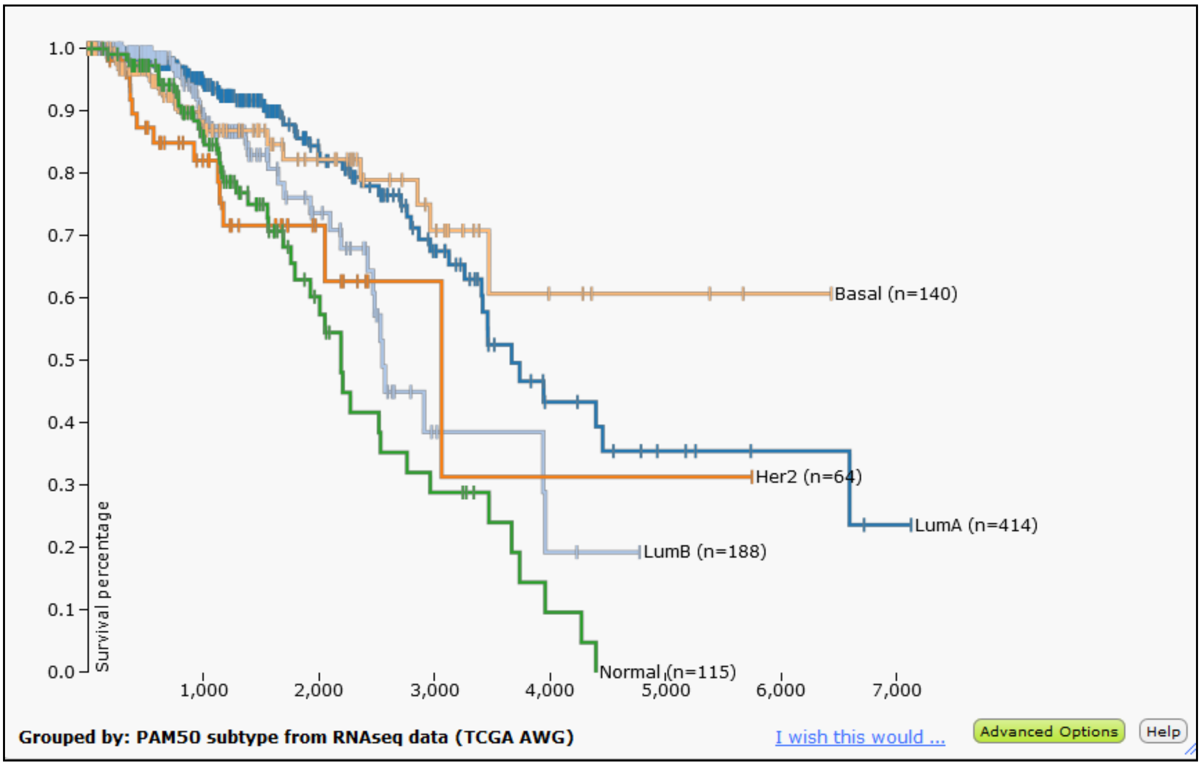

Survival analysis of TCGA dataset. Kaplan-Meier survival curves of breast cancer samples from the TCGA breast cancer RNA-Seq based database (UCSC browser was used for the analysis)

An additional observation was that if we take the list of genes with lowest p-values for association with prognosis from TCGA, this list is not enriched for immune related genes, as we observed in our data set and as it should be expected from recent literature, showing that immune infiltration is the among the most important prognostic factors in basal-like and triple-negative breast cancers.
On these bases we came to the conclusion that the survival data of the TCGA dataset present remarkable divergences from the majority of other data sets in the field [9], and that this probably explains the reasons for lack of convergence with our results. Based on these observations, it came by no surprise that also the exon-level results (EE and SI associated to prognosis) when compared between our and TCGA-based analyses present no overlap.

We therefore moved to the comparison with another public resource, accessible on-line (http://kmplot.com/analysis/). [10]. This database, here referred to as the KMP DB, is the largest public resource of standard Affymetrix-based gene expression data and clinical information from breast cancer, extracted from a large number of publically available studies (from GEO, TCGA and EGA). It encompasses data from 4,142 breast tumours, 54,675 Affymetrix probe set IDs and 70,632 gene symbols.

Out of the 204 genes associated with basal-like prognosis in our dataset (q-val < 0.1), 168 (83%) had q-val <0.1 in the KMP database (Fisher-test p-value of the overlap $< 10^{-20}$). As a negative control, when we took the 204 genes with lowest association with prognosis from our dataset, only 25% had a q-value < 0.1 in the KMP database.

We went on with the comparison of the list of highest and lowest genes associated with prognosis from the TCGA data set and those associated with prognosis in the KMP database, and we found a very limited overlap (30%). This was not significantly different from the overlap between the 204 genes with lowest association with prognosis from TCGA, used as a negative control (19%) (see Figure below).

In summary:

1. Gene-level comparison with the only data set publically available comprising exon-level expression and clinical level information (TCGA RNA-Seq breast cancer) showed no concordance. This data set presented remarkable deviations with respect to survival analysis from what expected from other published literature and data sets.

2. Conversely, gene-level comparison with the largest existing resource of standard Affymetrix-based gene expression data and clinical information – KMP - provided striking confirmation of our gene-level results of survival associated genes.

3. TCGA gene-level prognostic results were not overlapping with KMP-based ones, pointing again to the fact that this data presents peculiar survival features when compared to other published data sets.

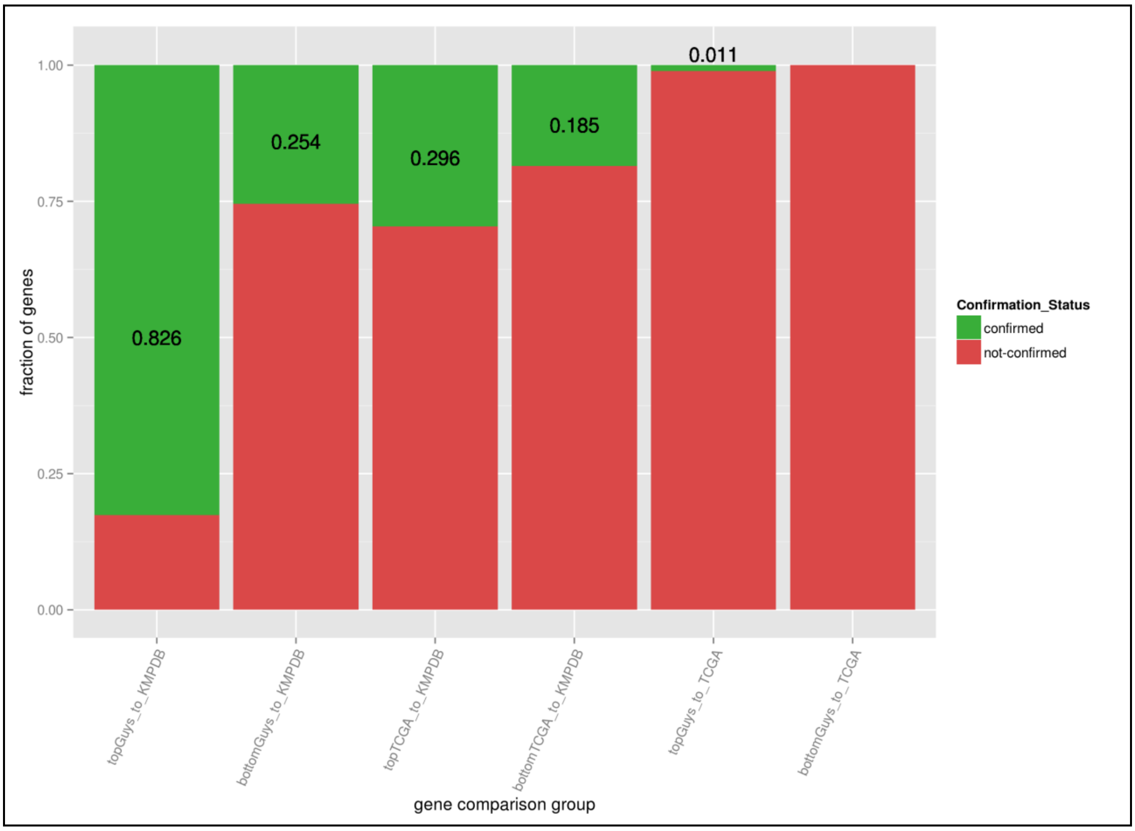

Comparison between basal-like breast cancer prognostic genes resulting from the analysis of our data set (Guy's), and two independent datasets (TCGA and KMP data sets). Each bar represents the fraction of genes identified as associated with survival in one dataset and checked in the second data set. From each database, q-value < 0.1 was set as a threshold for association with survival (Benjamini-Hochberg corrected p-value for multiple testing). Green and red colours respectively indicate presence confirmed and not-confirmed. From left to right: i) basal-like prognostic genes identified in Guy's and checked in KMPDB ii) basal-like non-prognostic genes identified in Guy's and checked in KMPDB iii) basal-like prognostic genes identified in TCGA and checked in KMPDB iv) basal-like non-prognostic genes identified in TCGA and checked in KMPDB v) basal-like prognostic genes identified in Guy's and checked in TCGA vi) basal-like non-prognostic genes identified in Guy's and checked in TCGA.

1. Sorlie T, Tibshirani R, Parker J, Hastie T, Marron JS, Nobel A, Deng S, Johnsen H, Pesich R, Geisler S *et al*: **Repeated observation of breast tumor subtypes in independent gene**

expression data sets. *Proceedings of the National Academy of Sciences of the United States of America* 2003, **100**(14):8418-8423.

2. Hu Z, Fan C, Oh DS, Marron JS, He X, Qaqish BF, Livasy C, Carey LA, Reynolds E, Dressler L *et al*: **The molecular portraits of breast tumors are conserved across microarray platforms**. *BMC Genomics* 2006, **7**:96.
3. Parker JS, Mullins M, Cheang MC, Leung S, Voduc D, Vickery T, Davies S, Fauron C, He X, Hu Z *et al*: **Supervised risk predictor of breast cancer based on intrinsic subtypes**. *J Clin Oncol* 2009, **27**(8):1160-1167.
4. Ignatiadis M, Singhal SK, Desmedt C, Haibe-Kains B, Criscitiello C, Andre F, Loi S, Piccart M, Michiels S, Sotiriou C: **Gene modules and response to neoadjuvant chemotherapy in breast cancer subtypes: a pooled analysis**. *J Clin Oncol* 2012, **30**(16):1996-2004.
5. Szasz AM, Lanczky A, Nagy A, Forster S, Hark K, Green JE, Boussioutas A, Busuttil R, Szabo A, Gyorffy B: **Cross-validation of survival associated biomarkers in gastric cancer using transcriptomic data of 1,065 patients**. *Oncotarget* 2016.

CYFIP2

Supplemental File 15

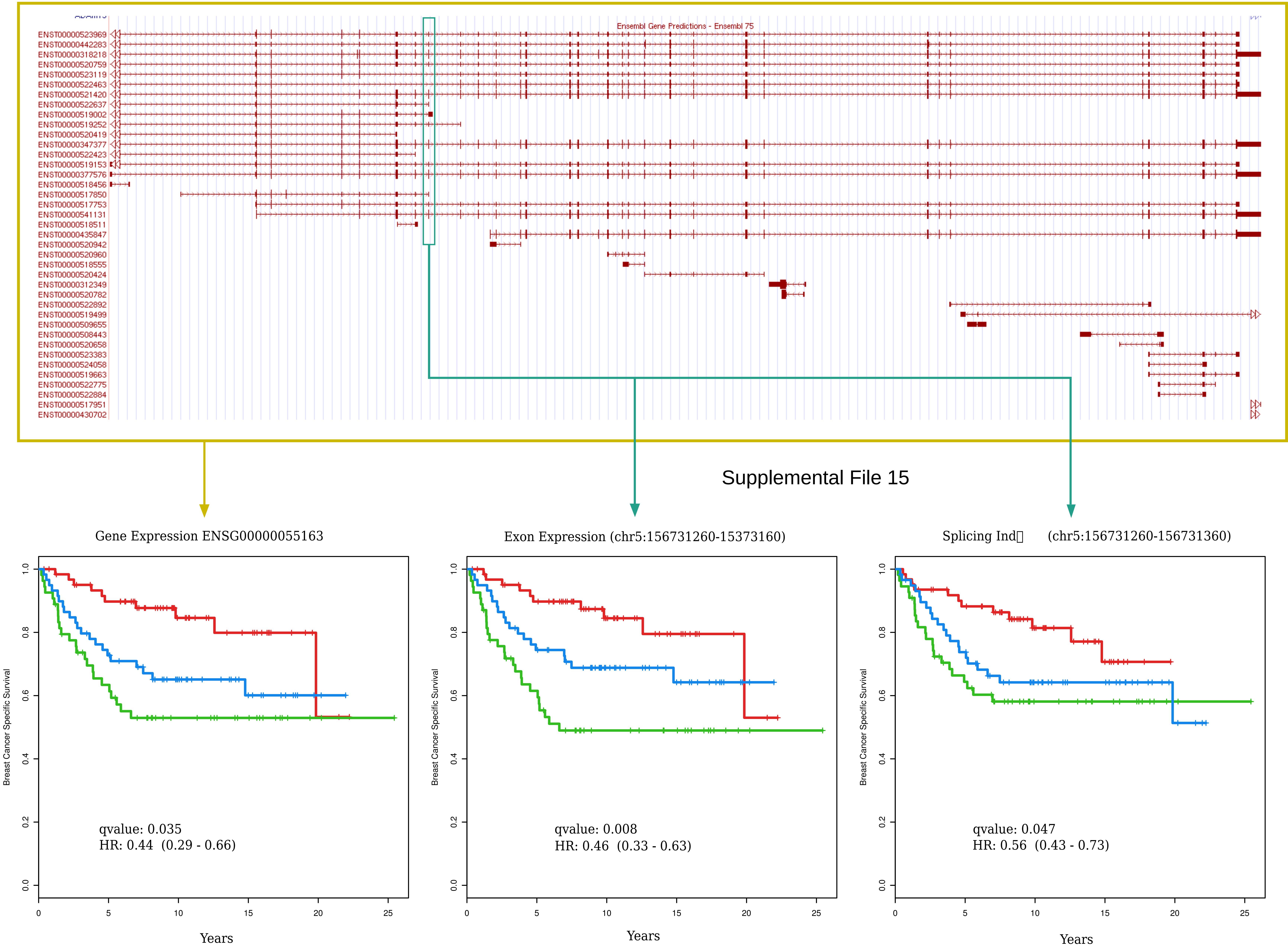

Supplemental File 16

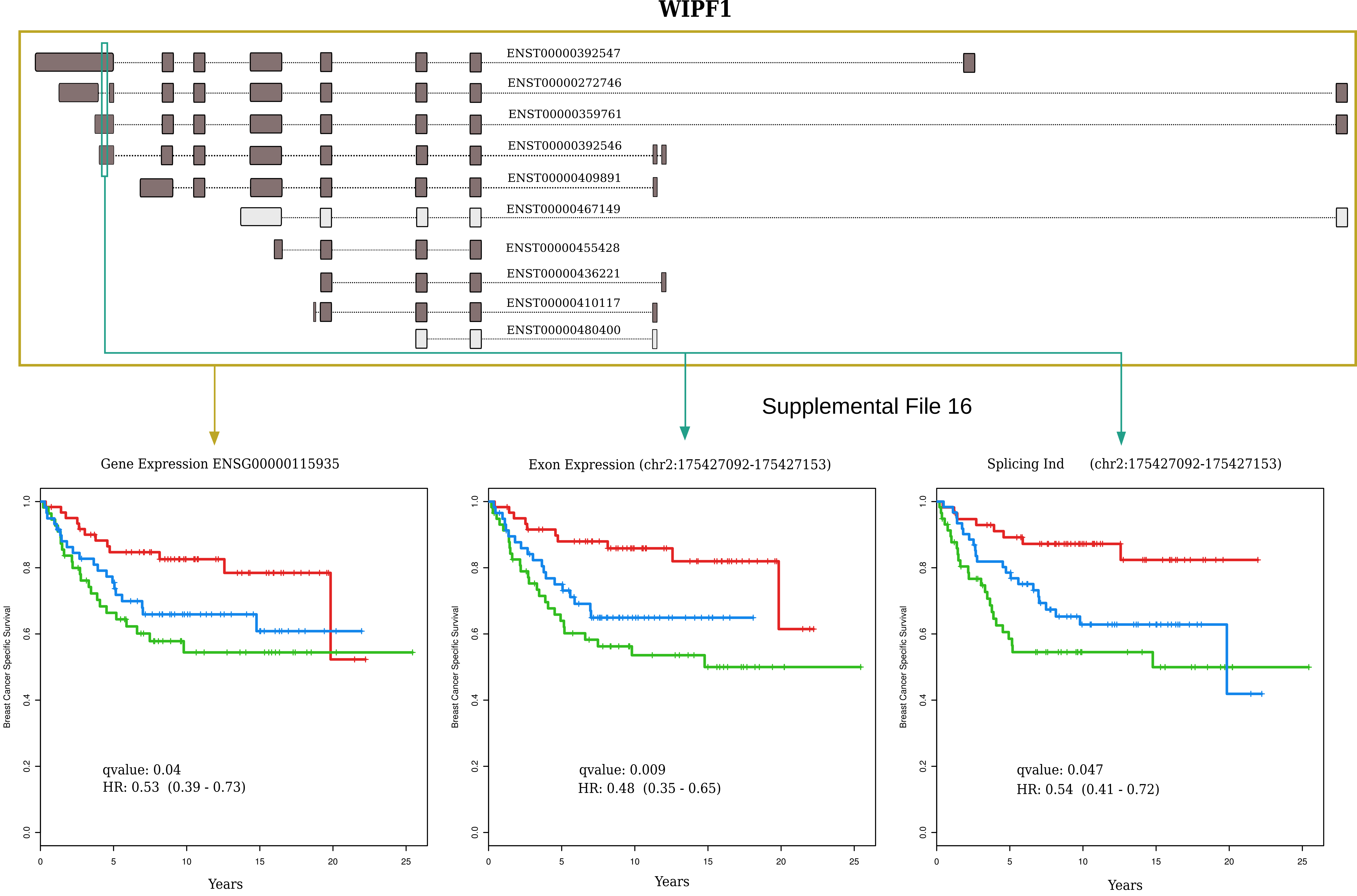

Supplemental File 17

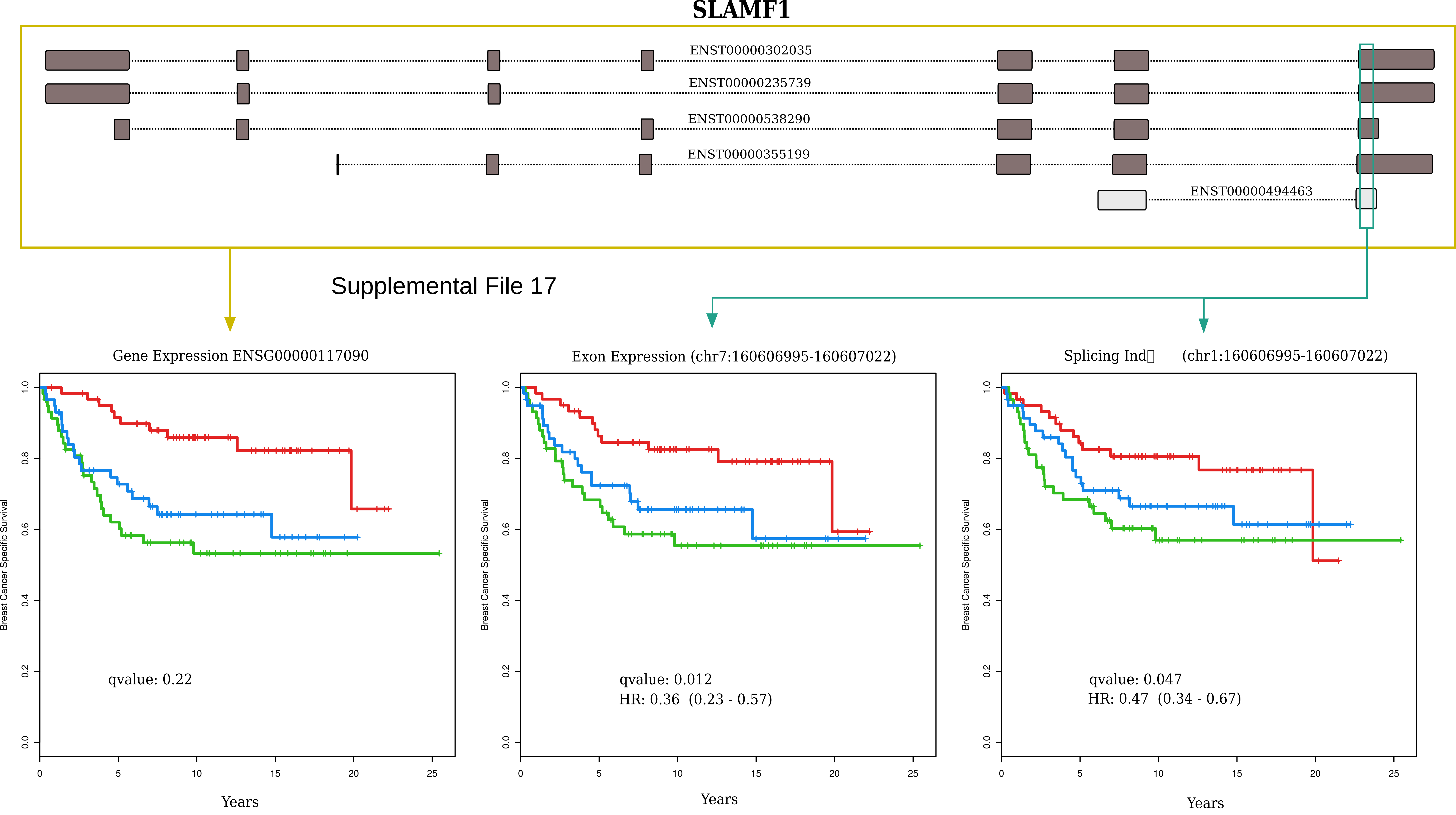

Supplemental File 18

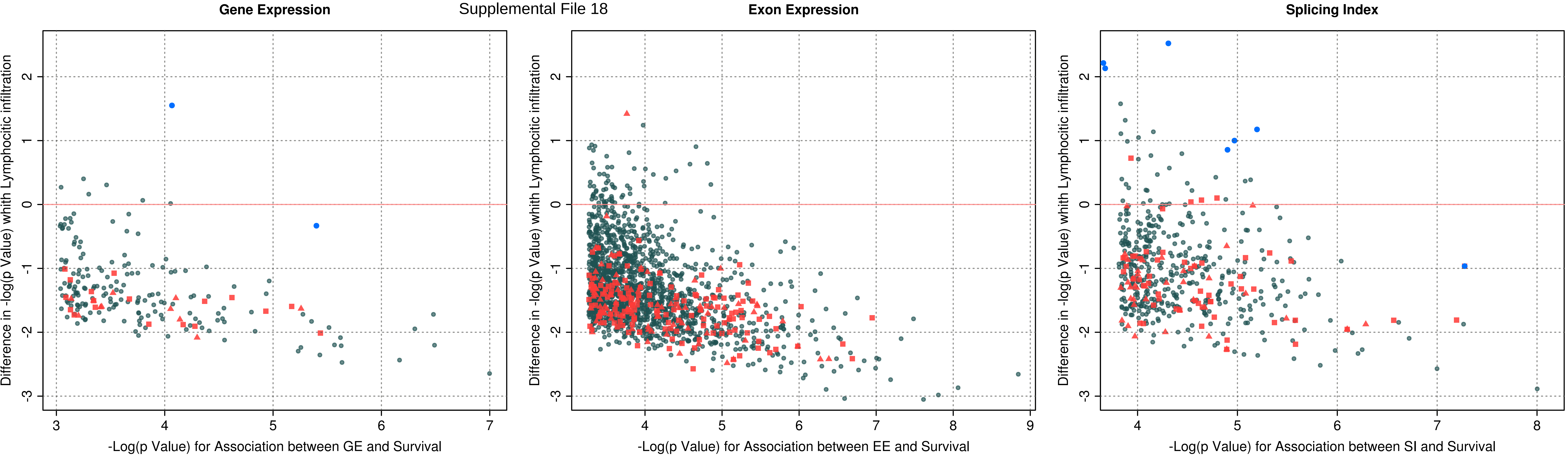

Exon Array Data Processing Workflow

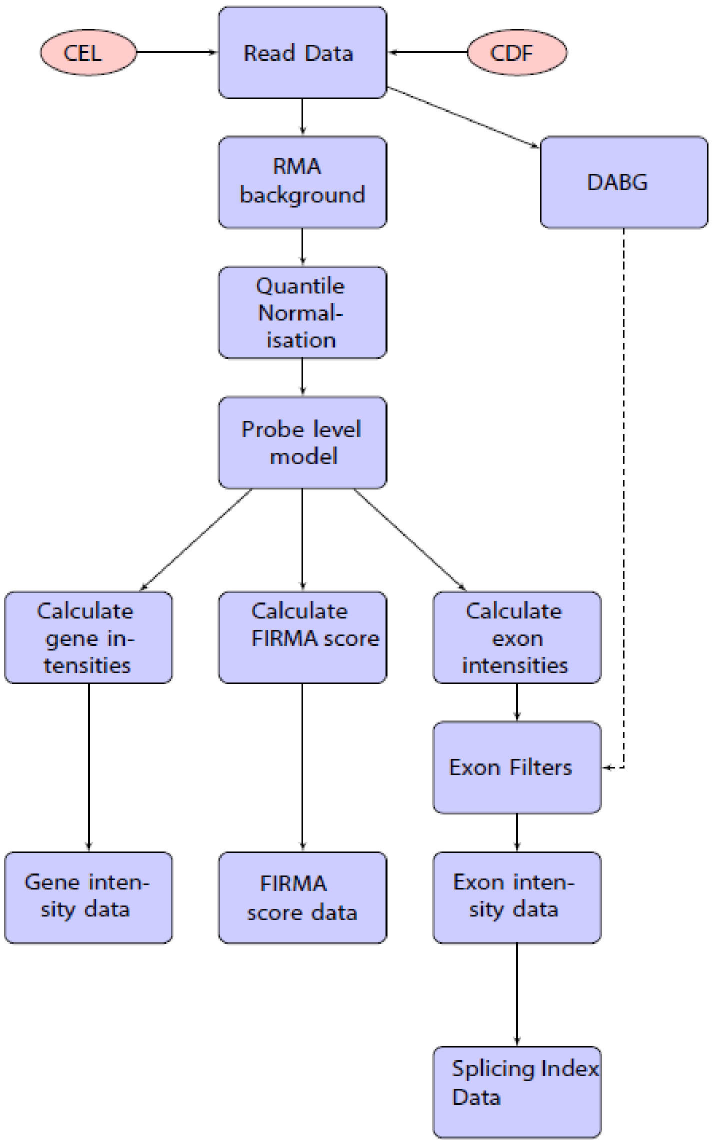

RLE plot

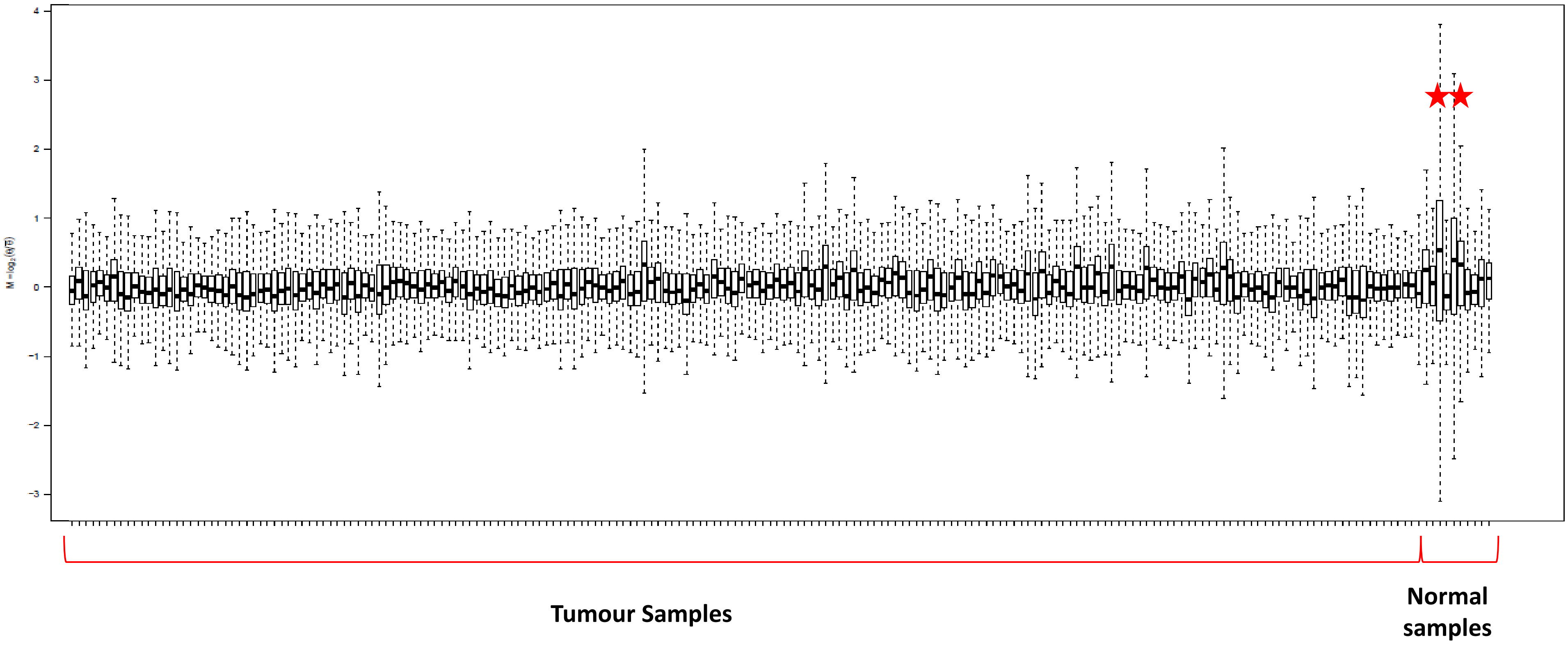

Relative Log Expression (RLE) values are computed by calculating for each probe-set the ratio between the expression of a probe-set and the median expression of this probe-set across all arrays of the experiment. The outlier normal samples highlighted by a red star were removed from the analysis

Hierarchical Clustering

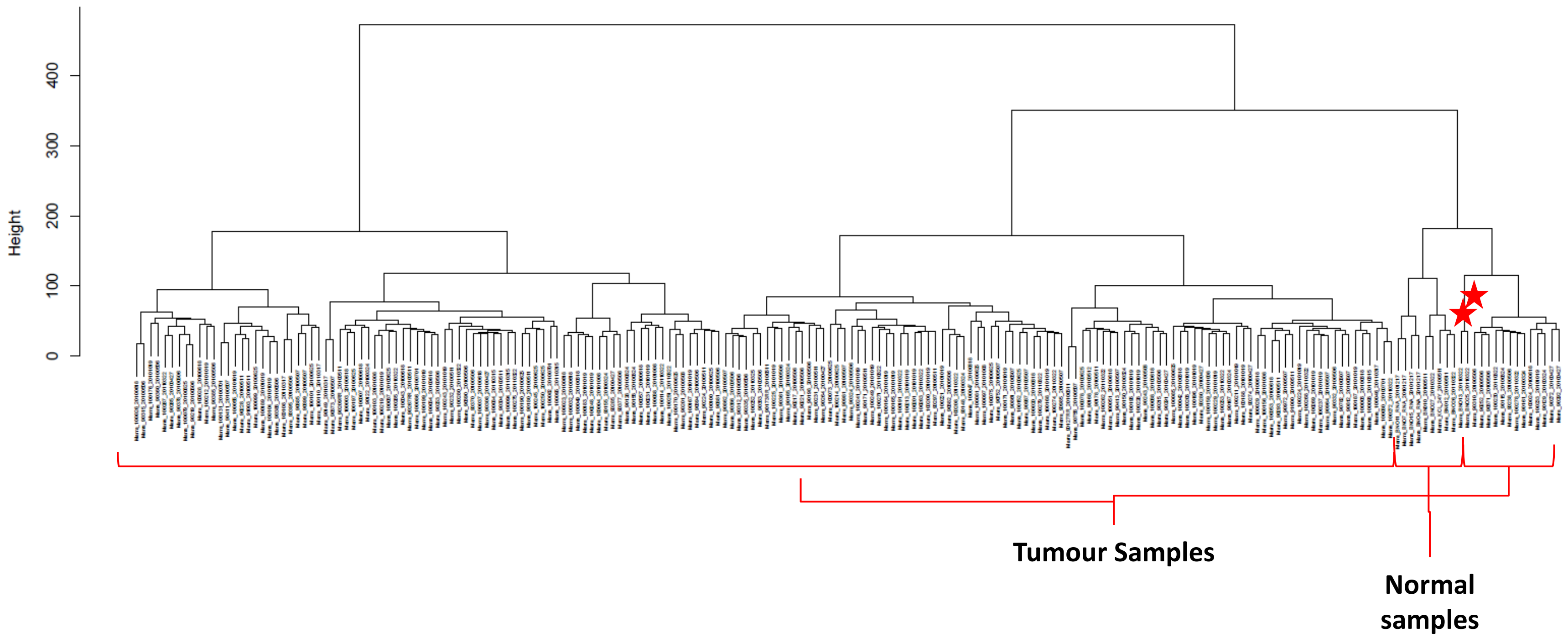

Hierarchical clustering of all samples was computed was computed after normalization. Two normal samples not clustering with the other normal samples (highlighted by red stars) were removed from the analysis

Description of additional data files

Description of additional data files

Additional File 1

Title: Sample phenotypic information.

Description: Sample_ID and Patient_ID: anonymized sample and patient IDs; SampleType: tumour or normal breast tissue; HER2, ER, PR: receptor status (P: positive, N: negative, NA: not available); Final_Grade: Tumour Grade; Percentage_of_Lymphocitic_cells: percentage of lymphocytic cells infiltrated in the Tumour; Diag_To_LastObs:number of days between first and last observation; AGE_at_DIAG: the patient's age at diagnosis. Surv_Status: the patient's status at last observation (1 deceased, 2 alive); PAM50: the sample PAM50 classification.

Additional File 2

Title: Intrinsic molecular subtypes and clinical markers.

Description: Characterization of different tumour types: PR, ER, HER2 receptor status according to IHC; histological grade; molecular intrinsic subtype assigned using transcriptional data and the PAM50 algorithm.

Additional File 3

Title: Coefficient of determination across sub types

Description: Distribution of R^2 values of each sample of a given group with all other samples of the same subtype (blue) or of different subtypes (red). From left to right, the three panels represent the results of the analysis using gene expression (GE), exon expression (EE) and splicing index (SI) values.

Additional File 4

Title: q-q plots of p-values from pairwise comparisons.

Description: Each of the panels shows the quantile-quantile plot for the distribution of the logarithm of p-values of a particular pairwise comparison against the logarithm of the uniform distribution. Each of the coloured lines represents different data types as indicated in the figure caption.

Additional File 5

Title: Classification Models.

Description: Sensitivity and Specificity plots for a classification model built to classify basal-like tumours and NBT. The horizontal axis represents the number of variables used in the model, the vertical axis represents sensitivity (in solid) and specificity (in dashed) measures. Different coloured lines refer to the different variables used. Variables were chosen at random from all genes/exons. More variables, naturally improve model quality.

Additional File 6

Title: Samples clustering using Principal Components Analysis (PCA).

Description: Principal components analysis on three types of data: gene expression (leftmost panel), exon expression (center panel) and splicing index (rightmost panel). Normal breast tissue samples are represented in red, and basal-like tumour samples in black. On the horizontal axis the second principal component, and on the vertical axis is the third principal component.

Additional File 7

Title: Comparison with independent studies

Description: Results of comparison between our results and three independent studies.

Additional File 8

Title: Experimental validation of differential splicing on 9 genes

Description: Description of the experimental protocol used and the results obtained for 9 genes differentially spliced in basal-like tumours vs normal samples or in basal-like tumours with good vs bad prognosis

Additional File 9

Title: Pathway Analysis Results

Description: 1st worksheet: Results from Ingenuity Pathway Analysis (IPA) for the set of genes only differentially spliced between basal-like and Normal Breast Tissue Ingenuity Canonical Pathways: the name of the pathway in the Ingenuity database. –log(p-value): the statistical significance of the enrichment as reported by Ingenuity. Ratio: the fraction of genes in the IPA pathway that overlapped the input list. Molecules: the names of the genes in the input gene list that are part of the IPA canonical pathway. 2nd and 3rd worksheets: p-values for gene set enrichment of differentially expressed or spliced genes against the gene sets of Molecular Signatures Database (MSigDb)

Additional File 10

Title: Paxillin Signalling Pathway

Description: The paxillin signalling pathway as represented by Ingenuity (Ingenuity® Systems). In purple are genes affected by differential splicing between basal-like tumours and normal breast tissues, with no evidence of whole-gene differential expression.

Additional File 11

Title: Gene sets perturbed at Splicing Index but not Gene Expression level – complete results.

Description: In this heatmap, each column is a pairwise comparison (either at GE or SI level), each row is a gene set and colour coded is the $log_{10 p\ value}$ for significant enrichment of that gene set for the list of differentially spliced genes for the respective pairwise comparison.

Additional File 12

Title: q-q plots of the p-values for association with Survival.

Description: Panels represent, from left to right, Gene Expression, Exon Expression, Splicing Index. Deviations from the diagonal (in red) indicate that the p-values differ from the theoretical uniform distribution expected for no association with survival.

Additional File 13

Title: Validation of survival analysis results using external data sets

Description: Document describing the rationale and the results obtained on gene expression and survival association from external datasets

Additional File 14.

Title: Results of Survival Analysis.

Description: This table contains all genes where either total expression or splicing index could be associated with survival in basal-like breast cancer. The 1st and 2nd worksheets contain respectively gene and exon level information. The 1st contains all the genes where at least one exon that could be associated with survival in basal-like breast cancer, either by their expression level, or by their splicing index (SI). It includes the Ensemble ID, the gene symbol, the coordinates of the probeset, the q-value and hazard ratios for association with survival in the one-factor model with EE, the q-value and hazard ratios for association with survival in the one-factor model with SI, the q value and hazard ratios for association with survival in the two-factor models (i.e. EE + lymphocytic infiltration, and SI + lymphocytic infiltration).

Additional File 15

Title: Kaplan–Meier curves for CYFIP2

Description: On the top: a schematic representation of the exon level gene model for CYFIP2 (taken from UCSC genome browser). On the bottom: the three panels, from left to right, show Kaplan-Meier survival curves for Gene Expression, Exon Expression, and Splicing Index. In each plot, the three lines represent the top tercile (red), middle tercile (blue), and lower tercile (green) for the value of the variable. In insert are the q-value for association with survival, and the hazard ratio with 95% confidence intervals.

Additional File 16

Title: Kaplan–Meier curves for WIPF1.

Description: On the top: a schematic representation of the exon level gene model for WIPF1 (not in scale). Dark grey represent protein coding isoforms, and light grey non-coding. On the bottom: the three panels, from left to right, show Kaplan-Meier survival curves for Gene Expression, Exon Expression, and Splicing Index. In each plot, the three lines represent the top tercile (red), middle tercile (blue), and lower tercile (green) for the value of the variable. In insert are the q-value for association with survival, and the hazard ratio with 95% confidence intervals.

Additional File 17

Title: Kaplan–Meier curves for SLAMF1

Description: On the top: a schematic representation of the exon level gene model for SLAMF1 (not in scale). Dark grey represent protein coding isoforms, and light grey non-coding. On the bottom: the three panels, from left to right, show Kaplan-Meier survival curves for Gene Expression, Exon Expression, and Splicing Index. In each plot, the three lines represent the top tercile (red), middle tercile (blue), and lower tercile (green) for the value of the variable. In insert are the q-value for association with survival, and the hazard ratio with 95% confidence intervals.

Additional File 18

Title: Effect of lymphocytic infiltration in survival multivariate model.

Description: Panels, from left to right, are for Gene Expression, Exon Expression, and Splicing Index. Each point in the plot is a gene or probe; the horizontal axis shows the log_{10pval} for association with survival in the univariate Cox Hazard model (GE, EE, or SI), and the vertical axis shows the difference in $log_{10ppval}$ for that gene/exon when lymphocytic infiltration is introduced as an additional covariate in the multivariate model. Negative values on this axis indicate loss of significance. Highlighted in red are genes related to lymphocytic function, as assessed either by gene expression or by database functional annotations (see Materials and Methods). Highlighted in blue are genes retaining statistical significance in the multivariate model.

Additional File 19

Title: Exon Array data processing analytical workflow and QCs

Description: Overview of the Exon Array data processing analytical workflow and results from microarray QC

Additional File 20

Title: Excel file with gene- and exon-level results of comparative analyses.

Description: q-values, and log fold change for statistically significant differences between sample groups (genes and exons showing no significance in any of the tests, were omitted from the table).
